# Comprehensive Antigenic Map of a Cleaved Soluble HIV-1 Envelope Trimer

**DOI:** 10.1371/journal.ppat.1004767

**Published:** 2015-03-25

**Authors:** Ronald Derking, Gabriel Ozorowski, Kwinten Sliepen, Anila Yasmeen, Albert Cupo, Jonathan L. Torres, Jean-Philippe Julien, Jeong Hyun Lee, Thijs van Montfort, Steven W. de Taeye, Mark Connors, Dennis R. Burton, Ian A. Wilson, Per-Johan Klasse, Andrew B. Ward, John P. Moore, Rogier W. Sanders

**Affiliations:** 1 Department of Medical Microbiology, Academic Medical Center, University of Amsterdam, Amsterdam, The Netherlands; 2 Department of Integrative Structural and Computational Biology, The Scripps Research Institute, La Jolla, California, United States of America; 3 Center for HIV/AIDS Vaccine Immunology and Immunogen Discovery, IAVI Neutralizing Antibody Center and the Collaboration for AIDS Vaccine Discovery (CAVD), The Scripps Research Institute, La Jolla, California, United States of America; 4 Department of Microbiology and Immunology, Weill Medical College of Cornell University, New York, New York, United States of America; 5 Program in Molecular Structure and Function, The Hospital for Sick Children Research Institute and Departments of Biochemistry and Immunology, University of Toronto, Toronto, Ontario, Canada; 6 HIV-Specific Immunity Section, Laboratory of Immunoregulation, National Institute of Allergy and Infectious Diseases, National Institutes of Health, Bethesda, Maryland, United States of America; 7 Department of Immunology and Microbial Science, The Scripps Research Institute, La Jolla, California, United States of America; 8 Ragon Institute of MGH, MIT, and Harvard, Cambridge, Massachusetts, United States of America; 9 The Skaggs Institute for Chemical Biology, The Scripps Research Institute, La Jolla, California, United States of America; Miller School of Medicine, UNITED STATES

## Abstract

The trimeric envelope (Env) spike is the focus of vaccine design efforts aimed at generating broadly neutralizing antibodies (bNAbs) to protect against HIV-1 infection. Three recent developments have facilitated a thorough investigation of the antigenic structure of the Env trimer: 1) the isolation of many bNAbs against multiple different epitopes; 2) the generation of a soluble trimer mimic, BG505 SOSIP.664 gp140, that expresses most bNAb epitopes; 3) facile binding assays involving the oriented immobilization of tagged trimers. Using these tools, we generated an antigenic map of the trimer by antibody cross-competition. Our analysis delineates three well-defined epitope clusters (CD4 binding site, quaternary V1V2 and Asn332-centered oligomannose patch) and new epitopes at the gp120-gp41 interface. It also identifies the relationships among these clusters. In addition to epitope overlap, we defined three more ways in which antibodies can cross-compete: steric competition from binding to proximal but non-overlapping epitopes (e.g., PGT151 inhibition of 8ANC195 binding); allosteric inhibition (e.g., PGT145 inhibition of 1NC9, 8ANC195, PGT151 and CD4 binding); and competition by reorientation of glycans (e.g., PGT135 inhibition of CD4bs bNAbs, and CD4bs bNAb inhibition of 8ANC195). We further demonstrate that bNAb binding can be complex, often affecting several other areas of the trimer surface beyond the epitope. This extensive analysis of the antigenic structure and the epitope interrelationships of the Env trimer should aid in design of both bNAb-based therapies and vaccines intended to induce bNAbs.

## Author Summary

The discovery of new broadly neutralizing antibodies against various epitopes on the HIV-1 envelope glycoprotein trimer and increased knowledge of its structure are guiding vaccine design. To increase our understanding of the interrelationships among the different epitopes, we generated a detailed antigenic map of the trimer using a variety of techniques. We have uncovered various mechanisms whereby antibodies can influence each other’s binding. The resulting antigenic map should further aid in design of HIV-1 vaccines to induce broadly neutralizing antibodies and in devising cocktails of such antibodies for therapeutic use.

## Introduction

The HIV-1 envelope glycoprotein (Env), a trimer comprising three gp120 and gp41 subunits, is the target of broadly neutralizing antibodies (bNAbs) that are known to prevent virus infection in animal models. The induction of bNAbs by vaccines is a highly desirable, but not yet achieved, goal. bNAbs isolated from HIV-1 infected individuals are templates for Env-based vaccines [1], and may also be useful as therapeutics [2]. Around 20% of infected people generate bNAbs [1]; their emergence usually takes at least 2 years, but can sometimes occur within a year [3].

Most bNAbs recognize epitopes in four well-defined clusters. They include PG9/16, PGT141-145, CH01-04 and VRC26 (gp120; quaternary structure-dependent V1V2-glycan), b12, VRC01, VRC03, PGV04, HJ16, CH31, CH103-106, 3BNC60, 3BNC117, 12A12, NIH45-46 (gp120; CD4 binding site; CD4bs), PGT121-123, PGT125-130, PGT135-137, 10–1074 (gp120; Asn332-centered oligomannose patch), and 2F5, 4E10, 10E8 (gp41; membrane-proximal external region; MPER) [reviewed in [1]], [4]. However, several bNAbs against new quaternary structure-dependent epitopes have now been isolated. The PGT151-158, 35O22 and 8ANC195 bNAbs interact with diverse epitopes at the gp120-gp41 interface [5–8]. Their quaternary structural requirements mean that they bind only, or far better, to soluble trimers that adopt a native-like conformation than to gp120 monomers or uncleaved, non-native gp140 proteins [5,6,8,9]. The 3BC315 bNAb was originally reported to target a CD4-induced gp120 epitope [10], but its epitope is now known to be on gp41 [11,12].

The precise relationships among the various bNAb epitope clusters on the Env trimer are not fully understood. An antibody cross-competition analysis helped to define the antigenicity of the gp120 subunit [13]. However, trimerization alters the conformation, surface accessibility and antigenicity of gp120, and hence many Abs that bind well to gp120 cannot recognize the trimer (i.e. non-neutralizing Abs; non-NAbs). Accordingly, we elected to conduct a comprehensive analysis of the antigenicity of bNAb epitopes on the trimer, and their relationships. The recent development of recombinant, soluble BG505 SOSIP.664 trimers that antigenically mimic native, virion-associated Env made this study possible [12,14–16]. These trimers express all epitopes for bNAbs that neutralize the parental virus, except those within the MPER [4–6,8,12,14,17–20], that are not included in the construct. Their high resolution structures in complexes with bNAbs PGV04 and PGT122 were solved by cryo-electron microscopy (EM) and X-ray crystallography, respectively [18,19], and a higher resolution X-ray structure of a complex with PGT122 and 35O22 is now available [21,22]. While the BG505 SOSIP.664 trimers are not identical to the native Env spike on viruses due to stabilizing mutations and truncation of the MPER they are excellent mimics based on extensive antigenic and structural data.

Here, we describe a comprehensive antigenicity analysis of the same trimers using an antibody cross-competition ELISA and a panel of bNAbs against all known epitope clusters (except MPER). Key ELISA-derived observations were corroborated and extended by surface plasmon resonance (SPR). We also prepared low resolution, negative stain EM reconstructions of the Env trimers in complex with CH103, CH106, 1NC9, 3BNC117 and VRC01. The new EM data, as well as published structures, allowed us to interpret the cross-competition data in a structural context and thereby create an antigenic map of the trimer surface. Using the full set of data, we found that bNAbs could inhibit one another’s binding by four different mechanisms: 1) epitope overlap, 2) steric inhibition, 3) allosteric inhibition, 4) glycan re-orientation. These findings enhance our understanding of how HIV-1 is neutralized, how the design of vaccines intended to induce bNAbs can be improved, and how bNAbs might be used therapeutically.

## Results and Discussion

### bNAb cross-competition ELISA

The goal of our cross-competition analysis was to characterize the interrelationships among bNAb epitopes on the trimer surface. In the assay, D7324-epitope-tagged, BG505 SOSIP.664 trimers [12] were immobilized on ELISA wells, an excess of non-biotinylated competitor bNAbs was incubated for 30 min with the immobilized trimers, and the biotinylated analyte bNAb was then added and its binding quantified (for pilot experiments, see S1A Fig). We selected 24 bNAbs against all known epitope clusters (other than the MPER), of which 19 could successfully be biotin-labeled. Five bNAbs (1NC9, 8ANC195, PGT151, 35O22, 3BC315) lost trimer reactivity after biotinylation presumably because key primary amines on lysine residues were modified. For those five bNAbs, we used Fabs as competitors for unlabeled IgG antibodies and detected the Fc-regions of the latter. We note that the use of Fabs might result in less steric competition compared to IgG, but, in vivo, the IgG competition is more relevant.

**Fig 1:**
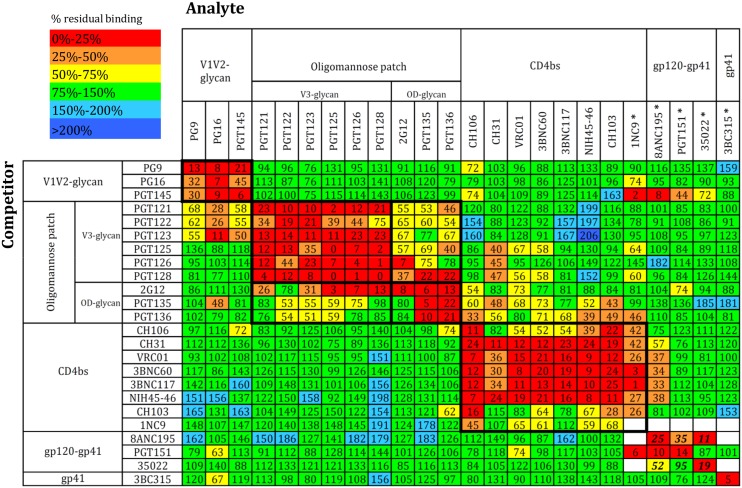
bNAb cross-competition matrix for the BG505 SOSIP.664 trimer. Competitors (IgG) are listed at the left and analytes (biotinylated IgG) at the top. Some bNAbs could not be biotinylated. In those cases (indicated by *), the competitors were Fabs and the analytes were IgG. The bNAb clusters were arranged according to their location on the Env spike from top to bottom (membrane-distal to membrane-proximal). Within clusters, the bNAbs were arranged in such a way as to obtain an optimal inhibitory effect along the diagonal axis. Color-coding depicts the extent of competition or enhancement: red and orange indicate strong and intermediate inhibition, yellow indicates weak inhibition and green indicates no effect. Weak and strong enhancement effects are indicated in turquoise and blue. The numbers inside each box are derived from 6–9 independent values and indicate the percentage of residual binding of the analyte relative to the control (100%). The standard errors for all data points are plotted in the accompanying matrix in S1*B* Fig. The 8ANC195 Fab was unable to self-compete, and the 1NC9 and 35O22 Fabs were only weakly self-competitive. The bold numbers for 8ANC195 and 35O22 represent competition values obtained by SPR, not ELISA, and are means of two replicates.

In the resulting cross-competition matrix, we plotted the percentage of residual bNAb binding in the presence of an excess of the competitor (Fig. 1). The EC_50_ values for each test antibody, and hence, the molar excess, in the competition are provided in S1 Table. The epitope clusters were arranged in the matrix from top-to-bottom according to how they are approximately oriented on the trimer: the quaternary, trimer-apex cluster is at the top, the oligomannose patch and CD4bs clusters are located lower down, and finally, the gp120-gp41_ECTO_ interface and the exclusively gp41_ECTO_ epitopes are at the bottom. The MPER epitopes at the base of the trimer were excluded because they are not present on the SOSIP.664 construct [12,23,24]. Note that the oligomannose patch is subdivided into V3-glycan and outer domain (OD)-glycan sub-clusters, based on the results of this study and reference [1]. Within each epitope cluster, the individual bNAbs were arranged according to their cross-competing properties. Self-competition, plotted along the diagonal, serves as a positive control and was generally very strong (<25% residual binding). Three exceptions were 1NC9, 8ANC195 and 35O22, for which Fabs served as competitors for complete IgGs (as noted, the biotin-labeled MAbs were non-reactive). We therefore excluded all ELISA data derived using these three Fabs, because they could not be properly interpreted. For 8ANC195 and 35O22, we instead used SPR (see below) to obtain information on the gp120-gp41_ECTO_ cluster.

### Gp120 epitope clusters

As expected, there were strong competitive effects between MAbs within each known epitope cluster. For example, within the V1V2-glycan trimer apex cluster, PG9, PG16 and PGT145 were strongly and reciprocally competitive.

The PGT121-123 and PGT125-128 family members all depend on the glycan at Asn332 and residues at the base of V3. All these bNAbs competed very efficiently with each other. The 2G12, PGT135 and PGT136 bNAbs also recognize the Asn332 glycan, but their epitopes also involve non-V3 components of the gp120 OD-glycan. 2G12 strongly competed with PGT135 and PGT136 but not *vice versa*, perhaps because PGT135 and PGT136 bind the trimer relatively poorly compared to 2G12 (>10-fold higher EC_50_) and so do not compete efficiently [12]. We saw moderate but consistent and mostly reciprocal competition between bNAbs from the oligomannose patch cluster, which is consistent with these antibodies all targeting a “supersite of vulnerability” centered around the Asn332 glycan, albeit in subtly different ways [20]. Thus, different bNAbs have distinct binding motifs on different sides of this glycan, and their angle of approach can also vary [20,25]. Notably, competition between the OD-glycan and V3-glycan sub-cluster members was not as strong as that seen within the two individual sub-clusters. The implication is that the “Asn332 supersite” does indeed contain two *bona fide* sub-sites of bNAb epitopes (Fig. 1).

Most bNAbs from the CD4bs epitope cluster competed strongly and reciprocally with each other. The binding of 1NC9, originally classified as a CD4bs-targeting bNAb [26] but not characterized extensively, was inhibited by every known CD4bs bNAb. This outcome supports the assignment of 1NC9 to the CD4bs cluster, but it also has some distinctive and unusual properties (see below).

### Gp120-gp41_ECTO_ interface epitopes

The PGT151, 35O22 and 8ANC195 bNAbs recognize three new epitopes at the gp120-gp41_ECTO_ interface [5,6,8,26]. As none of them retained trimer reactivity upon biotinylation, we used the Fab *vs*. IgG competition method. As noted above, self-competition was negligible for 8ANC195 and weak for 35O22, probably attributable to avidity issues; we therefore excluded all 8ANC195 and 35O22 Fab competition data from the matrix and instead relied on SPR (see below). In the competition ELISA, PGT151 Fab strongly inhibited 8ANC195 IgG binding to the trimer, which implies a substantial interrelationship between their epitopes. In contrast, the lack of competition between PGT151 and 35O22 suggests that these two epitopes overlap minimally or not at all. The 3BC315 bNAb did not cross-compete with any other antibody, which is consistent with its epitope being located on gp41. However, the lack of competition between 3BC315 and 35O22 was unexpected because the two epitopes overlap slightly [11].

### Interpreting off-diagonal competitions

The generally positive inhibition pattern along the diagonal axis of the matrix confirms existing knowledge of the test antibodies and their assignment to defined epitope clusters. In contrast, the off-diagonal inhibition patterns provide new clues about the physical proximity of, or functional relationships among, the different epitope clusters, and/or about the allosteric effects that arise when bNAbs bind the trimer.

The V3-glycan bNAbs PGT121, PGT122 and PGT123 inhibited binding of the V1V2-glycan cluster bNAbs (PG9, PG16, PGT145) relatively strongly, but in a non-reciprocal manner. PGT125, PGT126 and PGT128, also in the V3-glycan cluster, did not, however, block binding of the V1V2-glycan cluster bNAbs. The structure of the BG505 SOSIP.664 trimer complex with PGT122 illustrates that PG9, PG16 and PGT122 have slightly overlapping epitopes that all involve the Asn156 glycan [18]. We hypothesized then that steric occlusion via the constant domain of the Fabs also plays a role, providing a steric-based explanation for the cross-cluster competition. However, the non-reciprocal competition seen between, e.g., the PGT121-123 family and PG9, PG16 and PGT145 might be at least partially explained by the stoichiometry of binding. Thus, when three bNAbs of the PGT121-123 family (trimer stoichiometry = 3) are bound to the trimer, PG9, PG16 or PGT145 may have a very limited opportunity to gain access to their epitopes on its apex. However, when a single trimer-apex bNAb binds (trimer stoichiometry = 1), the PGT121-123 epitopes on one or two protomers could still be available for binding. Yet another possibility is that binding of PGT121-123 antibodies stabilizes the closed state of the trimer and limits the conformational flexibility required for the V1V2 apex antibodies to access their epitope on the trimer interface. Indeed, some bNAbs are now known to stabilize the closed conformation of Env [27,28]. We do not yet have an explanation for the strong, but non-reciprocal, competition between PG16 and PGT135.

The OD-glycan and CD4bs bNAbs competed to a moderate extent, but on a non-reciprocal basis (Fig. 1). A good example is that PGT135 and PGT136 inhibited the binding of most CD4bs bNAbs, but not *vice versa* (see below). We also observed competitive effects between bNAbs to the V3-glycan and CD4bs clusters. Thus, PGT125, PGT126 and PGT128, as well as PGT135 and PGT136, all inhibited CH31 binding; and PGT128 had the same non-reciprocal inhibitory effect on VRC01, an outcome that was confirmed by SPR (see below).

The binding of 8ANC195, which recognizes an epitope at the gp120-gp41_ECTO_ interface, was impeded by several CD4bs bNAbs (Fig. 1), suggesting some relationship between these two sites on the trimer.

The most striking off-diagonal inhibition pattern was the non-reciprocal competition between PGT145 and 1NC9, 8ANC195 and, to a lesser extent, PGT151 and 35O22. This outcome was surprising because these epitope clusters are not in close proximity on the trimer structure. The binding of PGT145, which has an absolute specificity for native trimers [9,12,29], to the apex may induce allosteric changes elsewhere in the trimer, or else stabilize the structure such that access to certain other epitopes is hampered by the reduced conformational flexibility. If so, the effect is highly specific to PGT145 as the same pattern was not seen using PG9 and PG16, which also recognize epitopes at the trimer apex. Any changes in the trimer induced by PGT145 binding were reversible, as PGT145- and 2G12- affinity purified BG505 SOSIP.664 trimers bound 1NC9 and PGT151 comparably (S2 Fig).

### Uni- and bidirectional binding enhancement

We found several examples of one bNAb enhancing the trimer binding of another, either reciprocally or uni-directionally. For example, the V3-glycan bNAb PGT128 bound more strongly in the presence of CD4bs bNAbs, 8ANC195 or 3BC315. There was reciprocally enhanced binding between the CD4bs bNAb NIH45-46 and the V3-glycan bNAbs PGT123 and PGT128; and between the gp120-gp41_ECTO_ bNAb 8ANC195 and the V3-glycan bNAb PGT126. Non-reciprocal enhancement between NIH45-46 with PGT121 and PGT123 was seen. The CD4bs bNAbs 3BNC117, NIH45-46, CH103 and 1NC9, and the gp120-gp41_ECTO_ interface bNAb 8ANC195 increased the binding of several V1V2-glycan, V3-glycan or OD-glycan bNAbs, for reasons that are currently under investigation.

### Competition by CD4 and induction of CD4-like conformational changes

**Fig 2:**
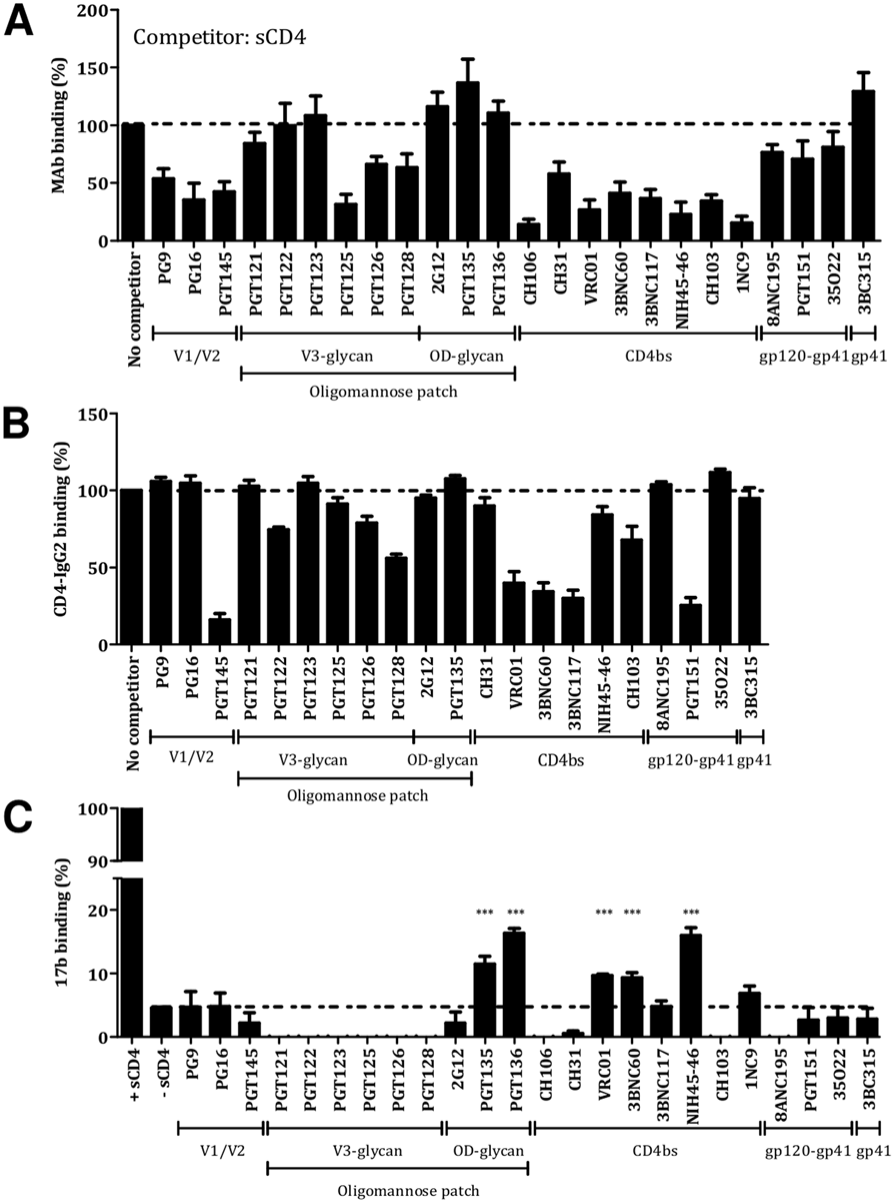
CD4 binding to the BG505 SOSIP.664 trimer and the induction of CD4-like conformational changes. (**A**) Use of sCD4 as a competitor. (**B**) Use of CD4-IgG2 as the analyte. (**C**) Induction of conformational changes measured by 17b binding. The extent of 17b binding in the presence of sCD4 was defined as 100%. *** indicates a significant difference between 17b binding without sCD4 and in the presence of a bNAb, as calculated using a Mann-Whitney 2-tailed test (P <0.05).

When we used soluble CD4 (sCD4) as the competitor ligand, we saw the expected strong inhibition of CD4bs bNAbs (Fig. 2A). The V1V2-glycan bNAbs also bound markedly less well in the presence of sCD4, which probably reflects how CD4 binding opens up the trimer apex and thereby impairs or eliminates the associated epitopes [30]. The binding of the V3-glycan bNAb PGT125 was also strongly inhibited, the family members PGT126 and PGT128 to a lesser extent. The results for PGT126 and PGT128 are consistent with observations made using sCD4, the V3-glycan bNAbs and monomeric gp120 [31].

Again as expected, Fabs of the various CD4bs bNAb strongly competed with CD4-IgG2 for trimer binding (Fig. 2B). The V1V2-glycan bNAb PGT145 also prevented CD4-IgG2 binding, consistent with its induction of long-range allosteric effects, while the V3-glycan bNAbs PGT122, PGT125, PGT126 and PGT128 markedly but incompletely inhibited CD4-IgG2. Finally, the gp120-gp41_ECTO_ interface bNAb PGT151 (but not the other two members of this cluster) strongly inhibited CD4-IgG2 binding. While the three bNAbs do all recognize gp120-gp41_ECTO_ interfacial regions, their epitopes are spatially separated [8]. The structural data on the PGT151-trimer complex is consistent with its inhibitory effect on CD4-IgG2 binding [5].

To investigate whether bNAbs could induce CD4-like conformational changes, we used the CD4i antibody 17b, a non-NAb, as a probe (Fig. 2C). The trimer binding of the CD4bs bNAbs VRC01, 3BNC60 and NIH45-46W increased the subsequent binding of 17b, although only to a modest extent (9% for VRC01 or 3BNC60, 16% for NIH45-46W, in a scale where sCD4 is 100%). Similarly to sCD4, VRC01 induces substantial conformational changes in monomeric gp120 that better expose the 17b CD4i epitope, but it differs from sCD4 in not having that effect on the functional, virion-associated Env trimer [32,33]. Hence the modest induction of the 17b epitope when VRC01 binds to the BG505 SOSIP.664 trimer (9% of the magnitude seen using sCD4) more closely reflects what is seen with native trimers than with monomeric gp120.

Of note is that the OD-glycan bNAbs PGT135 and PGT136 also enhanced 17b binding (by 12% for PGT135 and 16% for PGT136, again compared to 100% for sCD4), but no other bNAbs had this effect. Conversely, bNAbs from the V3-glycan cluster reduced the binding of both CD4i antibodies, which is consistent with the close proximity of the relevant epitopes on the trimer [34].

### Cross-competition studied using surface plasmon resonance

**Fig 3:**
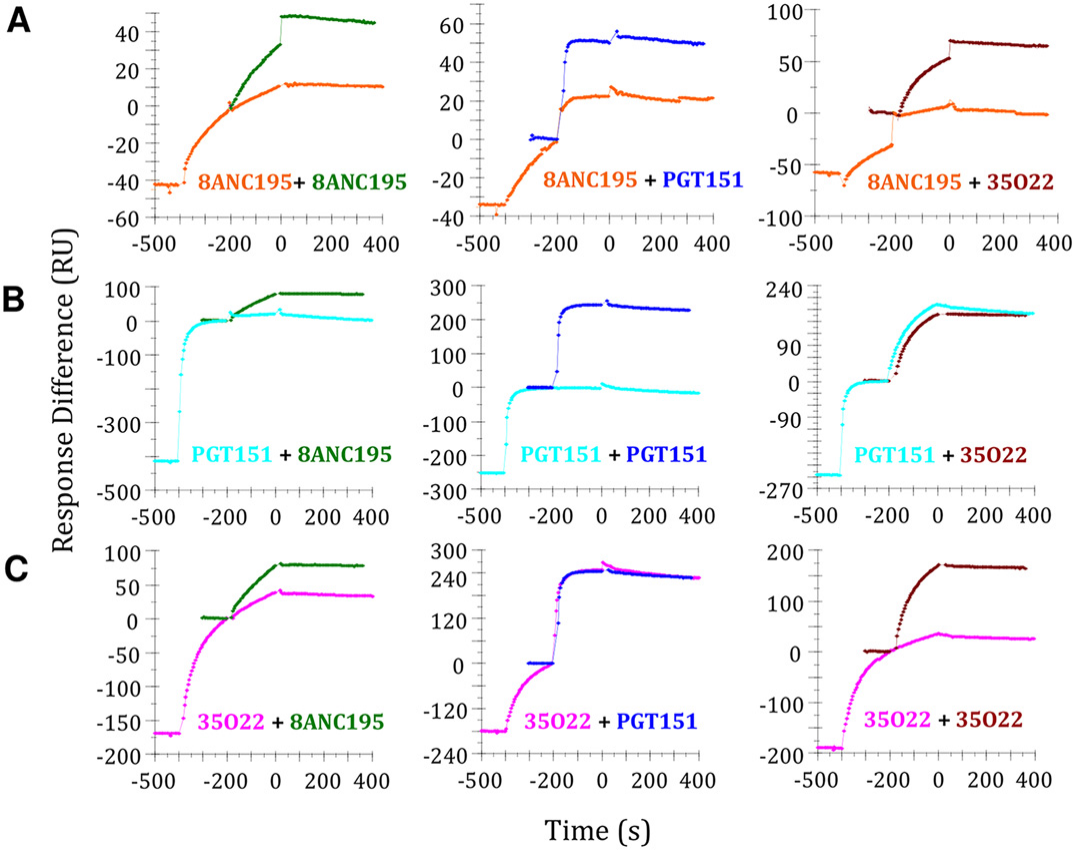
Cross-competition analysis for bNAb binding to BG505 SOSIP.664 trimer by SPR. (**A**) Competition between 8ANC195 (competitor) and PGT151 and 35O22. Association-dissociation curves of the individual binding experiments were overlaid with the second association phase to detect competition. 0 on the y axis is the baseline for the single comparator injection and for the same analyte as the second in the double injection. Thus, the strength of all three responses can be read on the same scale, although the value for the first analyte in the double injection will be negative. (**B**) Competition between PGT151 (competitor) and 8ANC195 and 35O22. (**C**) Competition between 35O22 (competitor) and 8ANC195 and PGT151.

We used SPR to further explore and extend selected ELISA-derived observations. His-tagged BG505 SOSIP.664 trimers were immobilized on the chip via an anti-His Ab, as previously described [35]. In a new SPR competition format, two analytes (NAbs) were sequentially injected in a single cycle, and the two association curves recorded, the dissociation curve only after the second injection. For comparison, the association-dissociation curves from the individual analyte injections were superimposed on the curve for the second injection of the same analyte. The resulting plots are interpreted as follows: If the second curve for the sequential binding superimposes well on the individual binding curve there is no competition, whereas if the individual-injection curve is higher than the curve for the second injection in the sequential binding, there is competition. The conditions were adjusted such that the self-competition was nearly complete (Fig. 3).

We were particularly interested in bNAbs 8ANC195, PGT151 and 35O22 against the gp120-gp41_ECTO_ interface because the corresponding ELISA datasets for 8ANC195 and 35O22 were incomplete. In the SPR assay, 8ANC195, PGT151 and 35O22 all showed strong self-competition (Fig. 3). When PGT151 and 35O22 were added to the 8ANC195-trimer complex, strong competition was again seen (Fig. 3A, middle and right panel). The same was true when 8ANC195 was added to the PGT151-SOSIP.664 complex, which is consistent with the competition seen by ELISA (Fig. 3B, left panel). However, 35O22 did not compete with the PGT151-SOSIP.664 complex, which is again consistent with ELISA data (Fig. 3B, right panel and Fig. 1). There was bidirectional competition between 8ANC195 and 35O22 for trimer binding in the SPR format but, in contrast, PGT151 bound efficiently to the 35O22-trimer complex (Fig. 3C, middle panel).

Overall, the SPR data show that 8ANC195 cross-competes reciprocally with both PGT151 and 35O22, whereas PGT151 and 35O22 do not cross-compete with each other. The implication is that 8ANC195 binds the trimer at a location between the PGT151 and 35O22 epitopes. A few other selected ELISA-derived observations were also checked using SPR; with only minor and generally subtle exceptions, the two datasets were in good agreement (S3 Fig).

### Structural interpretation of bNAb cross-competitions

To gain more insight into the relationships among bNAb epitopes in the trimer context, we used existing low resolution structures of several bNAb complexes with the BG505 SOSIP.664 trimer [5,8,12,14,17,18,20,36]. We also prepared similar *de novo* reconstructions of these trimers in complexes with CD4bs bNAbs CH103, CH106, 1NC9, 3BNC117 and VRC01. The 2D class averages and 3D reconstructions show all five antibodies recognize the trimers in similar ways, although with important differences (S4, S5A Figs). Cross-correlation coefficient analyses of the 3D map fitting show that CH103 and CH106 have an almost identical angle of approach that is different from those taken by VRC01, 1NC9 and 3BNC117 (S5B Fig). The 3BNC117 and 1NC9 bNAbs have a higher cross-correlation coefficient with VRC01 than do CH103 or CH106, suggesting there are subtle differences in the epitopes of these pairs of antibodies. Docking the CH103 crystal structure into the EM map indicates that gp120 has rotated relative to the ground state, and that the trimer is now in a slightly more open conformation. A study of several bNAbs using BG505 SOSIP.664 mutant trimers revealed that the 1NC9 and 3BNC117 epitopes differ slightly from those for more typical CD4bs bNAbs (i.e., VRC01, PGV04, CH103) by their dependence on the Asn276 glycan (S7 Fig).

**Fig 4:**
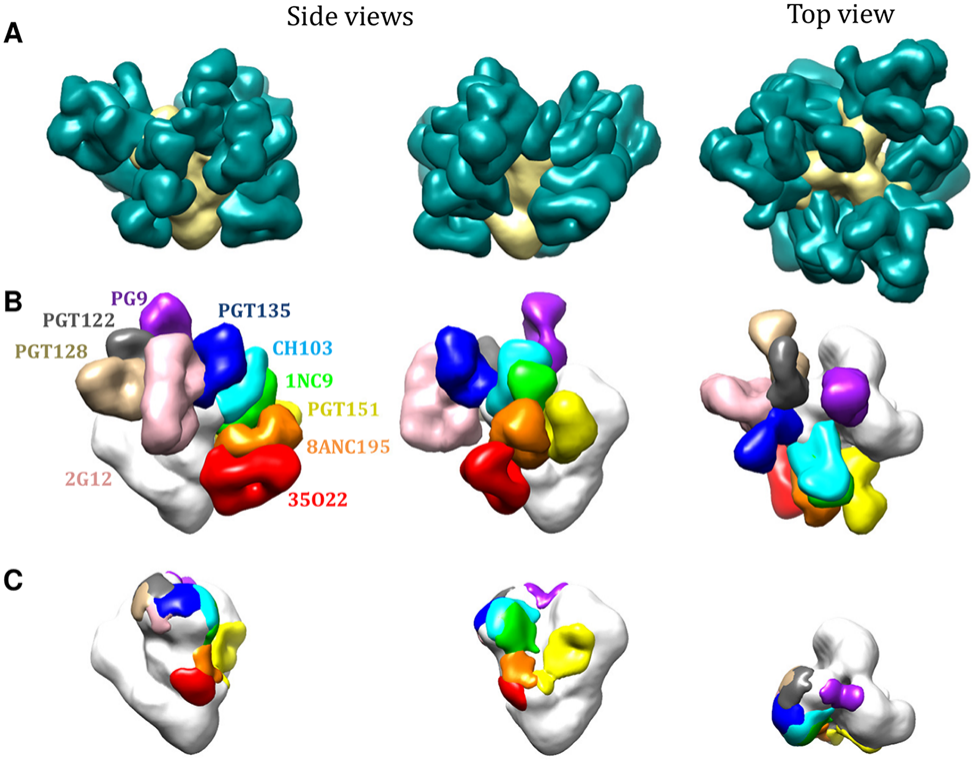
bNAb epitopes mapped onto the 3D structure of the BG505 SOSIP.664 trimer. (**A**) Various bNAbs (not labeled) to different epitope clusters are modeled onto each protomer of the trimer, according to fitting of various EM density maps (see material and methods). (**B**) bNAbs to different epitope clusters are modeled onto the same EM density map. Only one Fab fragment per trimer is shown for clarity. (**C**) Footprints of the different bNAb Fab fragment densities displayed in (**B**). See also methods for further details.

**Fig 5:**
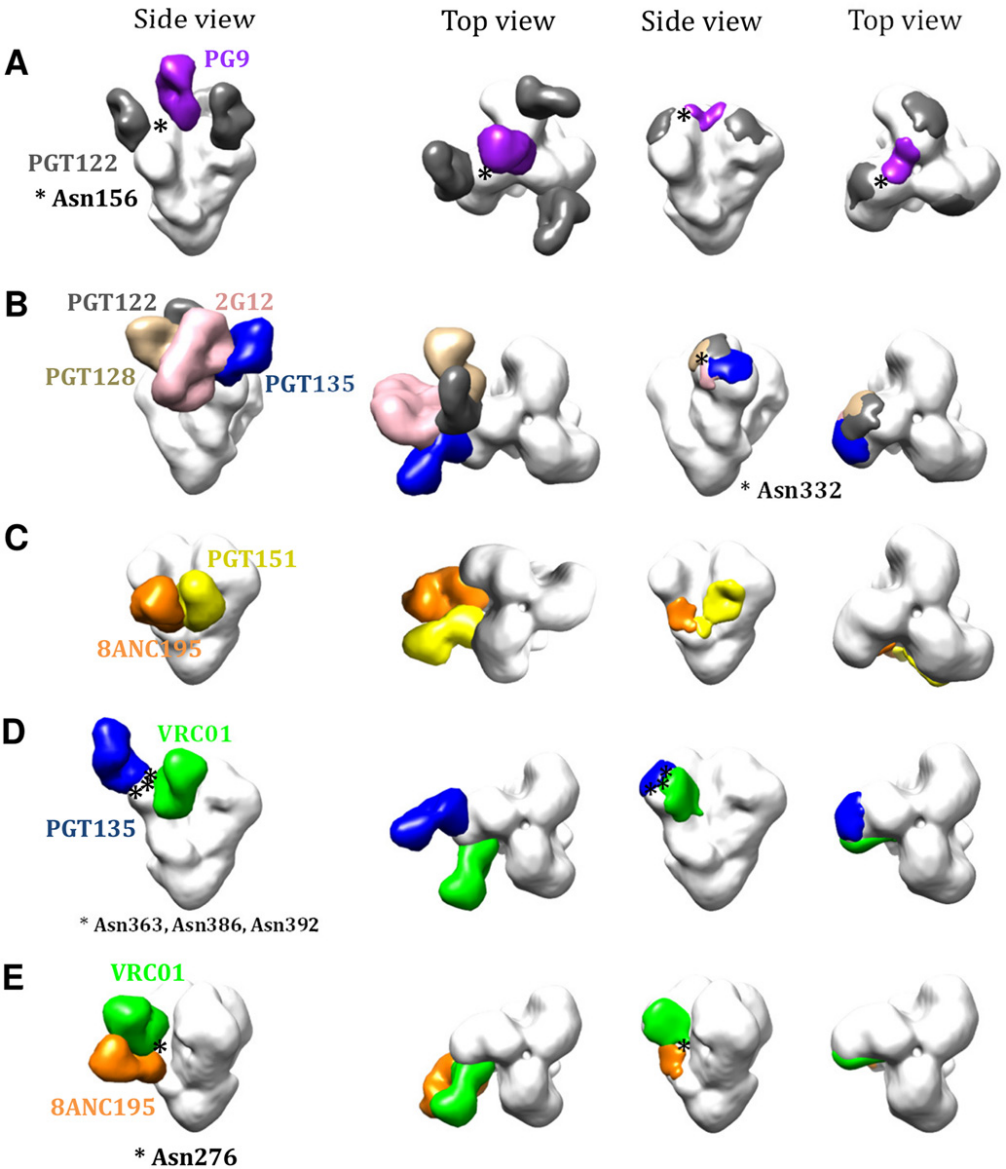
3D modeling to explain bNAb competition patterns. (**A**) 3D models for the nonreciprocal competition between PG9 and PGT122. Side and top views of the bNAbs are shown together with the footprints. (**B**) Analysis of the Asn332 supersite depicting the angles of approach to the Asn332 glycan taken by various bNAb subfamilies. (**C**) 3D models to explain the bidirectional competition between PGT151 and 8ANC195. (**D**) 3D models to explain the unidirectional competition between OD-glycan and CD4bs bNAbs and (**E**) 3D models to explain the nonreciprocal competition between 8ANC195 and CD4bs bNAbs.

Combining new and published EM data allowed us to assess how bNAbs interact with the trimer, yielding a qualitative estimate that approximately 60% of the trimer surface is covered by at least one known bNAb (Fig. 4A). When the various epitope clusters and the footprints of individual bNAbs were modeled onto the 3D trimer structure (Fig. 4B, C), the resulting models help to explain several outcomes of the cross-competition analysis. For example, we saw nonreciprocal competition between the V1V2-glycan bNAbs (e.g. PG9) and the V3-glycan bNAbs (e.g. PGT122) (Fig. 1). The 3D model indicates that PG9 and PGT122 have slightly overlapping epitopes centered around Asn156 (Fig. 5A) [18]. The model also confirms why the competition between these two bNAbs is unidirectional (i.e., PGT122 impedes PG9, but not the converse); the outcome arises from the binding stoichiometry considerations referred to above.

Inspection of the Asn332 supersite shows the various angles of approach to this glycan taken by the different bNAb families (Fig. 5B). While PGT122 and PGT128 approach the glycan from the same side, they do so at slightly different angles, whereas PGT135 and 2G12 approach from the opposite side. As a result, almost no component other than the Asn322 glycan is shared by the PGT122/PGT128 and PGT135/2G12 epitopes. The structural data are, therefore, consistent with the variable competition between the bNAbs from the V3-glycan (PGT122, PGT128) and OD-glycan (2G12, PGT135) sub-clusters (Fig. 1).

PGT151 competed strongly and reciprocally with 8ANC195. However, although both bNAbs target the gp120-gp41_ECTO_ interface, their epitopes do not overlap [5,36]. Modeling both Abs onto the 3D image of the trimer now shows that the competition between PGT151 and 8ANC195 is rooted in steric hindrance that arises from clashes in the constant region of the Fabs; i.e., the epitopes are sufficiently close to one another that both bNAbs cannot gain access to the trimer simultaneously (Fig. 5C).

### bNAb cross-competition by glycan reorientation

**Fig 6:**
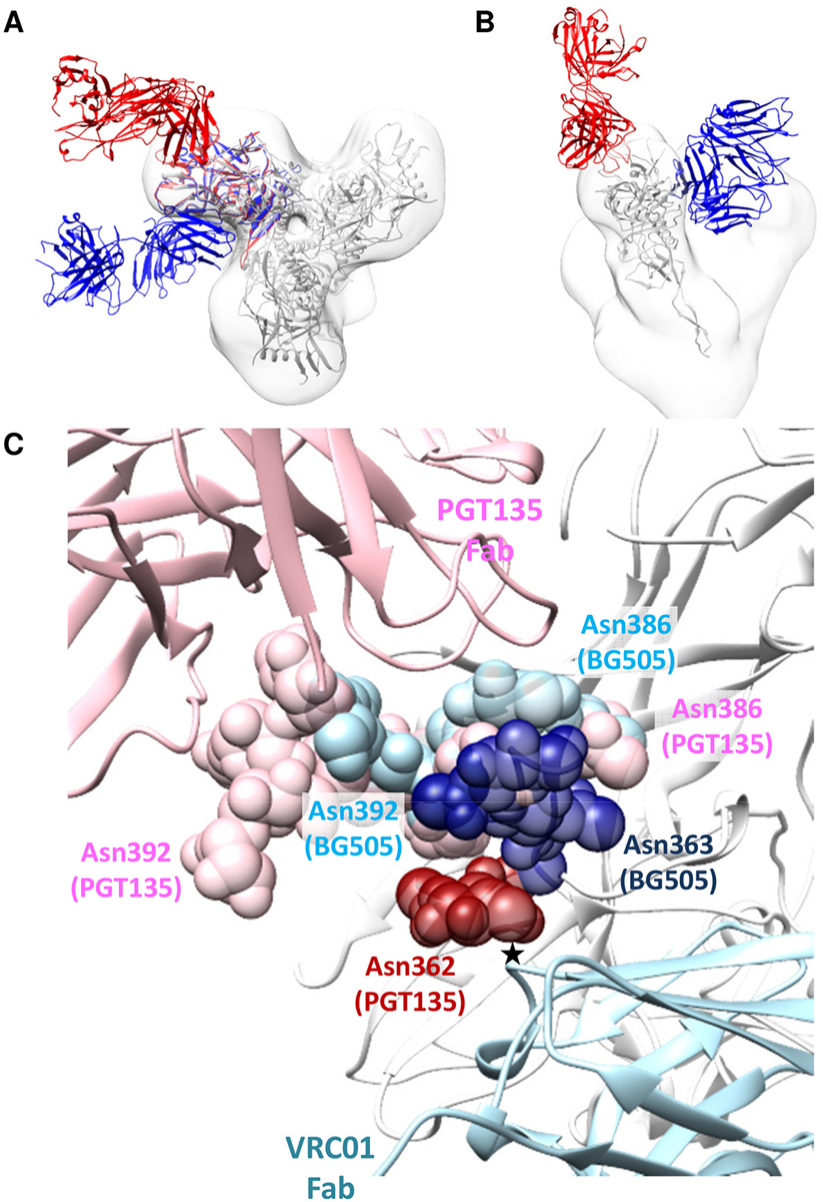
Comparison of PGT135 and VRC01 binding to HIV-1 gp120. **(A)** View down the trimer three-fold axis showing a superimposition of PGT135 Fab in complex with the clade B JRFL gp120 core (PDB ID: 4jm2; red), and of VRC01 Fab in complex with the clade A/E 93TH057 gp120 core (PDB ID: 3ngb; blue), each aligned onto the crystal structure of the BG505 SOSIP.664 trimer (PDB ID: 4tvp; white). In addition, the same BG505 SOSIP.664 crystal structure is fit into the negative-stain EM reconstruction of JR-FL Env trimer in complex with PGT151 (EMD-5919; white). The resulting model clearly shows that PGT135 and VRC01 do not sterically block binding of each other. **(B)** Side view of **(A)** with only one gp120 monomer displayed for clarity. **(C)** Detailed view of the key glycans (spheres) that were resolved in the PGT135-gp120 structure, and of the same glycans from the BG505 SOSIP.664 crystal structure. The presumed steric clash between glycan on Asn362 (and perhaps also Asn363) and the CDR H2 loop of VRC01 is marked with an asterisk. The gp120 subunit of the BG505 SOSIP.664 trimer is displayed as white ribbons. The glycans depicted were limited to the components resolved in the crystal structures (GlncNAc_1_ for Asn362, GlncNAc_2_Man_1_ for Asn386 and GlncNAc_2_Man_6_ for Asn392 in the PGT135-gp120 structure; GlncNAc_2_ for Asn362, GlncNAc_2_ for Asn386 and GlncNAc_2_ for Asn392 in the BG505 SOSIP.664 trimer structure), and hence their sizes are underestimated compared to trimers produced in 293F cells or present on viruses.

One unexpected example of nonreciprocal competition involved bNAbs from the CD4bs and OD-glycan clusters. Furthermore, binding inhibition could sometimes be seen even when the competitor bNAb bound the trimer only comparatively weakly in the ELISA. Specifically, based on their EC_50_ values, the OD-glycan bNAbs PGT135 and PGT136 bound ~5-fold less efficiently than the CD4bs bNAbs VRC01, 3BNC60, 3BNC117 and NIH45-46 [12], yet the former were still able to substantially, and unidirectionally, inhibit the binding of the latter (Fig. 1). Previous epitope mapping results from electron microscopy and x-ray crystallography studies clearly show that these two epitope clusters are independent and non-overlapping (Fig. 5D and Fig. 6A and B). We hypothesized that the decreased binding of CD4bs antibodies after PGT135 attachment is not caused by steric hindrance of the Fabs themselves, but by rearrangement of either the gp120 subunit or one or more specific glycan structures. The relative positions of the Fabs also agree well with negative-stain EM maps of the same Fab-trimer complexes (EMD-2331 [20]). Previous reports that the CDR H1 and H3 loops of PGT135 Fab interact closely with gp120 glycans Asn386 and Asn392 [20] are consistent with the Env trimer crystal structure (Fig. 6C). After superimposing the VRC01-gp120 structure onto the Env trimer structure, it appears that, when PGT135 binds to the trimer, the penetration of its CDR H1 loop into the glycan canopy would push the Asn363 glycan, and to a lesser extent the Asn386 glycan, toward the CD4bs. This movement would then create a steric clash between the Asn363 glycan and the CDR H2 loop of VRC01 (Fig. 6C) [37]. In contrast, a similar analysis indicates that VRC01 binding to the trimer does not cause significant movement of the Asn386 and Asn392 glycans into positions where they could sterically impede PGT135 binding. It should be noted that the crystal structures were derived using gp120s and trimers produced in 293S cells and EndoH-treated to remove as much glycan as possible prior to crystal formation. Hence only the residual, truncated glycan components that were resolved in the crystal structures were modeled. The Asn363, Asn386 and Asn392 glycan structures on the 293F cell derived trimers used in the competition ELISAs (and on viruses) will be substantially larger and the potential for clashes accordingly greater. When we performed similar docking analyses using the cryo-EM structure of 293F cell-derived trimers in complex with PGV04, additional density could be seen to extend from Asn363 that passed over the top of the VRC01 Fab (PGV04 and VRC01 have very similar, overlapping epitopes; EMD-5779 [19]). This suggests that the Asn363 glycan may have a limited range of conformations that are permissive for binding of CD4bs antibodies.

The unexpected nonreciprocal competition between 8ANC195 and CD4bs bNAbs, particularly VRC01, 3BNC60, 3BNC117 and NIH45-46, can also now be explained on structural grounds. The 8ANC195 epitope spans both the gp120 and gp41_ECTO_ subunits but is dependent on the Asn276 glycan that borders the membrane-proximal side of the CD4bs (Fig. 5E). Deleting this glycan increases the neutralization potency of VRC01 but, when present, the VRC01 light chain binds to it [38]. We hypothesize that when VRC01, 3BNC60, 3BNC117 or NIH45-46 bind the trimer, the Asn276 glycan is reoriented in a way that 8ANC195 can no longer recognize it. However, because Asn276 is not an critical component of the epitopes for some (e.g., VRC01), but not all (i.e., 3BNC117) CD4bs bNAbs [39] (S7B Fig), the competition is non-reciprocal. The enhanced binding of 3BNC117 by 8ANC195 may be related to dependency of Asn276 for 3BNC117 binding. Thus, 8ANC195 might reorient this glycan in such a way that it becomes more accessible to 3BNC117. Other CD4bs bNAbs such as CH103 and CH106 bind to the membrane-distal side of the CD4bs and have a steeper angle of approach than VRC01; these antibodies probably do not reorient the Asn276 glycan in a way that precludes 8ANC195 binding (Fig. 5E).

### Conclusions

**Fig 7:**
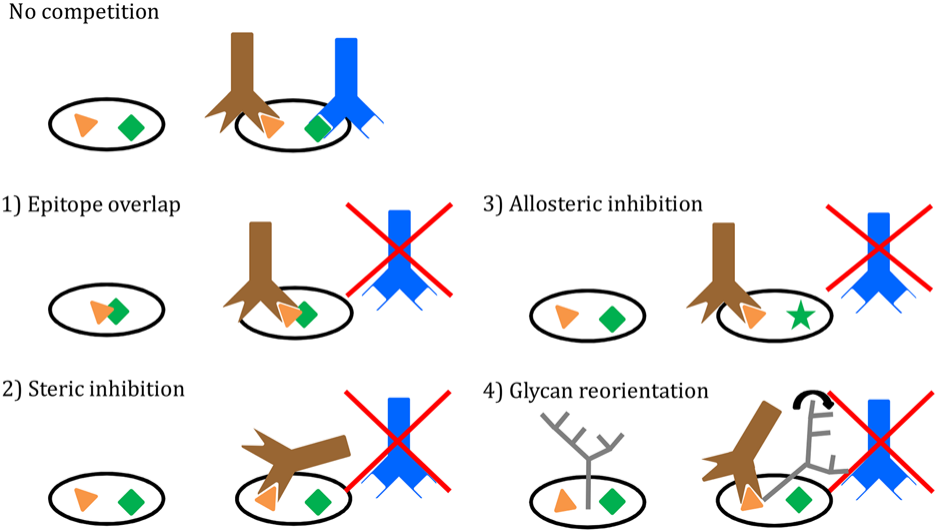
Four mechanisms of bNAb binding interference. (**1**) Direct overlap of epitopes; (**2**) Steric inhibition; (**3**) Allosteric inhibition; (**4**) Reorientation of glycans. See text for details.

Using a bNAb cross-competition ELISA, supported by SPR data and structural analyses, we have defined the steric and allosteric relationships among the known antigenic sites on the HIV-1 Env trimer. We have also identified four mechanisms by which bNAbs can interfere with one another’s binding (Fig. 7): 1) direct overlap of epitopes (many examples); 2) steric inhibition (PGT151 inhibition of 8ANC195); 3) allosteric inhibition (PGT145 inhibition of 1NC9, 8ANC195, PGT151 or CD4); 4) glycan reorientation (PGT135/PGT136 inhibition of CD4bs bNAbs, and CD4bs bNAb inhibition of 8ANC195). Whether competition between bNAbs is or is not reciprocal can also be influenced by both binding affinity (competition in ELISA is likely to be influenced substantially by bNAb off-rates) and stoichiometry (whether 1, 2 or 3 copies of a bNAb bind the trimer). Overall, based on our current observations, the binding of bNAbs to the trimer can involve local remodeling of loops or glycans, as well as triggering long-distance allosteric effects.

The platform for our experiments is the soluble, recombinant BG505 SOSIP.664 trimer, a highly stable native Env spike mimetic. While we are confident that these soluble trimers closely resemble the native spike, as shown by the strong correlation between antibody binding in ELISA and neutralization of the corresponding virus [12], differences may need to be considered when comparing an engineered stabilized soluble protein versus the wild-type membrane-associated protein. However, we suggest that such differences are more likely to be manifested at the qualitative than the quantitative level, for example in the rates and extent of antibody-induced conformational changes to the trimer and any subsequent allosteric effects.

One seemingly common belief about the trimer until recently was that it has only a few sites of vulnerability. That view emerged in a period when not many bNAbs were known [40–43]. A re-evaluation now seems to be warranted. The availability of entirely new families of bNAbs and identification of their epitopes on the structure of the trimer reveals many weaknesses in Env’s defenses against antibodies. Additional novel bNAbs are still likely to be isolated that may expose more. Thus, instead of just a few distinct sites of vulnerability there are many; and they overlap with one another. Computational modeling what is now known about the antigenic surface of the trimer allows one to take a virtual walk over the trail of partially overlapping epitopes that span the entire trimer from top to bottom. An example of the route mapped out from apex-to-base involves the following epitopes PG9-PGT122-PGT128-2G12-PGT135-CH103-1NC9-8ANC195-35O22 (Fig. 4B and C; Fig. 5). The overlap between these sites suggests that there is an almost continuous antigenic surface that can be recognized by one or more known bNAbs. Key questions now might be: does the human immune system target some vulnerable areas more frequently, or more effectively, than others? Does the development of an antibody response against one epitope influence the development of antibodies against other sites and, if so, how [44,45]?

The existence of indirect interference mechanisms for bNAb binding has implications for the use of competition ELISAs, or similar assays, for mapping the NAb/Ab specificities in sera from Env-immunized animals or HIV-1-infected people. For example, if an anti-Env serum inhibited the trimer binding of a CD4bs bNAb such as VRC01, it would not necessarily mean that VRC01-like antibodies were present in the serum as the inhibitory effect could be indirect [46].

Our detailed antigenic map of the BG505 SOSIP.664 trimer may assist in the design of vaccines aimed at inducing bNAbs, and for mapping responses in sera from infected individuals or from recipients of Env vaccines. This information may also be useful for designing cocktails of bNAbs for therapeutic use. For example, only antibodies with mutually reinforcing and not competing properties should be used together. Finally, the competition matrix can be considered the benchmark for analysis of where newly discovered bNAbs target the Env trimer.

## Materials and Methods

### Env trimers

The design of BG505 SOSIP.664 trimers, including the D7324-epitope tagged version, has been described extensively elsewhere, as have the methods to produce and purify them [12,14,18,19]. 2G12-affinity and size exclusion chromatography (SEC)-purified BG505 SOSIP.664-D7324 and SOSIP.664-His trimers were used for the ELISAs and SPR, respectively. For EM reconstructions, 2G12-affinity and SEC-purified BG505 SOSIP.664 trimers without the D7324-epitope tag were used. In the experiments in S2 Fig, BG505 SOSIP.664-D7324 trimers were purified via PGT145-affinity chromatography without SEC.

### Antibodies and Fabs

MAbs were obtained as gifts, or purchased, or expressed from plasmids, from the following sources directly or through the AIDS Reagents Reference Program: John Mascola and Peter Kwong (VRC01); Polymun Scientific (2G12); Michel Nussenzweig (3BNC60, 3BNC117, NIH45-46, NIH45-46W, 1NC9, 8ANC195, 3BC315); Barton Haynes (CH31, CH103, CH106,); James Robinson (17b). MAbs were biotin labelled using the Pierce EZ-Link Sulfo-NHS-Biotinylation kit (product code #21425) and Fabs were produced with the Pierce Fab purification kit (product code #44685), in both cases according to the manufacturer’s instructions.

### D7324-capture ELISA using BG505 SOSIP.664 trimers

The D7324-capture ELISA has been described elsewhere [12]. For the detection of biotinylated MAbs, horseradish peroxidase (HRP)-labeled streptavidin (Sanquin, The Netherlands) was used.

### D7324-capture competition assay using BG505 SOSIP.664 trimers

Microlon-600 96-well, half-area plates (Greiner Bio-One, Alphen aan den Rijn, The Netherlands) were coated overnight with Ab D7324 (Aalto Bioreagents, Dublin, Ireland) at 10 μg/ml in 0.1 M NaHCO_3_, pH 8.6 (50 μl/well). After washing and blocking steps, purified, D7324-tagged BG505 SOSIP.664 trimers were added at 300 ng/ml in TBS/10% FCS for 2 h. A 25-μl aliquot of TBS (150 mM NaCl, 20 mM Tris) plus 2% skimmed milk containing the competitor MAbs/Fabs (10 μg/ml of each in a 50 μl final volume) was added to each well of a separate plate. Unbound Env proteins were washed away from the test plate before the competitor MAbs were added and incubated for 30 min. A 25-μl aliquot of the biotinylated MAbs/IgG, at a concentration giving 50–70% of the maximum binding signal, was then added for 2 h followed by 3 washes with TBS. HRP-labeled streptavidin (Sanquin, The Netherlands) or HRP-labeled Goat anti-Human IgG, Fcγ fragment specific (Jackson Immunoresearch, Suffolk, England) were added for 1 h at a 1:3000 dilution in TBS/2% skimmed milk, followed by 5 washes with TBS/0.05% Tween-20. Colorimetric detection was performed using a solution containing 1% 3,3′,5,5′-tetramethylbenzidine (Sigma-Aldrich, Zwijndrecht, The Netherlands), 0.01% H_2_O_2_, 100 mM sodium acetate and 100 mM citric acid. Color development was stopped using 0.8 M H_2_SO_4_ when appropriate, and absorption was measured at 450 nm.

### Surface Plasmon Resonance

An anti-histidine antibody (GE Healthcare Bio-Sciences) was immobilized onto CM5 chips by amine coupling. His-tagged BG505 SOSIP.665 trimers were captured in amounts corresponding to 500 RU except in experiments with 8ANC195 in which 100 RU as used because of the NAb's slow association. Also PGT151 and 35O22 were used at 500nM, 8ANC195 at 1.5uM. SPR was performed under conditions previously described with modifications as follows [35]. In a new SPR competition format, two analytes (NAbs) were sequentially injected in a single cycle. The first analyte was injected and then after 200 s of the association phase, the second analyte was injected, also for 200 s, both at flow rates of 30 μl/min. After the second injection, dissociation was followed for 400 s. For comparison, each analyte was also injected on its own at the indicated concentrations. After each complete cycle, the chip surface was regenerated with a single pulse of 10mM Glycine (pH2.0) for 120s at a flow rate of 30 μl/min.

### Electron microscopy

BG505 SOSIP.664 gp140 trimers in complex with Fabs were analyzed by negative stain EM after overnight incubation at room temperature (6 molar excess of Fab to trimer). A 3 μL aliquot containing ~0.03 mg/mL of the Fab-trimer complex was applied for 5 s onto a carbon-coated 400 Cu mesh grid that had been glow discharged at 20 mA for 30 s, then negatively stained with 2% (w/v) Uranyl formate for 60 s. Data were collected using an FEI Tecnai T12 electron microscope operating at 120 keV, with an electron dose of ~25 e^-^/Å^2^ and a magnification of 52,000x that resulted in a pixel size of 2.05Å at the specimen plane. Images were acquired with a Tietz TemCam-F416 CMOS camera using a nominal defocus range of 1000 nm.

### Image processing and 3D reconstruction

Data processing methods were adapted from those used previously [9,12]. Particles were picked automatically using DoG Picker and put into a particle stack using the Appion software package [47]. Initial, reference-free, two-dimensional (2D) class averages were calculated using particles binned by two via Iterative MSA/MRA and sorted into classes [48]. Particles corresponding to complexes were selected into a substack and binned by two before another round of reference-free alignment was carried out using the Iterative MSA/MRA and Xmipp Clustering and 2D alignment software systems [49].

For Fab-containing complexes, an *ab initio* common lines model was calculated from reference-free 2D class averages in EMAN2 [50] using 3-fold symmetry. EMAN [51] was used for all 3D reconstructions, and all maps were refined using 3-fold symmetry. In total, 39,259 particles were included in the final reconstruction for the 3D average of BG505 SOSIP.664 trimer complex with CH103; 27,144 for the complex with CH106; 11,235 particles for the complex with 1NC9; 14,956 for the complex with 3BNC117; and 7,861 for the complex with VRC01.

To estimate the percentage of the HIV-1 Env surface covered by bnAbs, various EM models [PGT128 (EMDB 1970), PGT122 (EMDB 5624), PG9 (EMDB PG9), PGT135 (EMDB 2331), 2G12 (EMDB 5982), PGV04 (EMDB 5779), PGT151 (EMDB 5918), 8ANC195 (EMDB 2625) and 35O22 (EMDB 2672)] were loaded into the Chimera software package [52]. These models were then fit into the map of full-length JR-FL Env (i.e., including the transmembrane region) in complex with PGT151 (EMDB 5918) after removal of the PGT151 density. The contact area between various Fab densities and the trimer was estimated and the sum divided by the total surface area of the trimer. The surface area of each bNAb footprint is an estimate based on docking Fab X-ray structures into their respective EM reconstructions, and then coloring a zone on the Env surface that is within 4 Å of the Fab atoms. The 2G12 Fab footprint is colored within 8 Å of the docked Fab structure because it binds to the tips of several high mannose glycans that are not resolved in the low-resolution model. To account for surface area at the bottom of the trimer that is inaccessible due to the cell/virus membrane, the trimer volume was segmented and the surface area for a small portion in the transmembrane region was subtracted from the total trimer surface area. In addition, the contact area between 2G12 and the trimer was doubled to allow a more accurate measurement, taking into account that a gap between the Fab and trimer densities in the EM model arises from the high-mannose glycan patch (including the 2G12 epitope) not being resolved in the negative-stain reconstruction.

## Supporting Information

S1 Table Concentrations and binding parameters of the bNAbs in the cross-competition assay. Midpoint binding titers (EC_50_ values; second column) are taken from reference [12]. The analyte and competitor concentrations used here are given in the third and fourth column, and the molar ratio between the analyte and competitor antibodies are listed in the last column. Note that the molar ratios for Fab competitors (see asterisks) were halved to reflect the monovalency of the combining site compared to IgG.
(TIF)

S1 Fig bNAb cross-competition pilot experiment and standard error (%) of the cross-competition matrix. (**A**) Detection of biotinylated versions of 2G12 or NIH45-46 in the presence of escalating amounts of IgG competitors. (**B**) The extent of the error between individual data points is depicted by color-coding: Green, orange and red indicate small, intermediate and large standard errors between the individual data points, respectively.
(TIF)

S2 Fig ELISA using 2G12/SEC- and PGT145-purified BG505 SOSIP.664 trimers to determine whether allosteric changes induced by PGT145 were reversible. The 2G12 bNAb was used as a loading control and 1NC9 and PGT151 were used to probe the allosteric changes are still present in the PGT145 purified trimers. Because the binding of 1NC9 and PGT151 to 1G12/SEC- and PGT145-purified trimer is similar, we infer that the PGT145-induced allosteric changes that inhibit 1NC9 and PGT151 binding (Fig. 1) are reversible.
(TIF)

S3 Fig Cross-competition analysis for bNAb binding to BG505 SOSIP.664 trimer by SPR. (**A**) Competitions between PG16, PGT128 and VRC01. Association-dissociation curves of the individual binding experiments were overlaid with the second association phase to detect competition. 0 on the y axis is the baseline for the single comparator injection and for the same analyte as the second in the double injection. Thus, all three responses can be read on the same scale, although the value for the first analyte in the double injection will be negative. (**B**) Competition between PGT121 and VRC01.
(TIF)

S4 Fig Negative stain EM data of CH103, CH106, 1NC9, 3BNC117 and VRC01 Fabs in complex with BG505 SOSIP.664 trimers. Shown are the 2D class-averages of complexes of the trimers with (**A**) CH103 Fabs; (**B**) CH106 Fabs; (**C**) 1NC9 Fabs; (**D**) 3BNC117 Fabs; and (**E**) VRC01 Fabs. For reconstructions of the unliganded BG505 SOSIP.664 trimer, see references [12,14].
(TIF)

S5 Fig 3D-reconstruction of CH103, CH106, 1NC9, 3BNC117 and VRC01 in complex with BG505 SOSIP.664 trimers. (**A**) EM reconstruction of trimers in complex with CH103, CH106, 1NC9 3BNC117 and VRC01 Fabs, with a fit of the CH103 complex map into the 1NC9 complex map shown at center. (**B**) Cross-correlation coefficients from map fitting of the four BG505 SOSIP.664-Fab complexes.
(TIF)

S6 Fig Fourier shell correlation curves for BG505 SOSIP.664-Fab complexes (**A**) BG505 SOSIP.664 in complex with CH103 Fab (FSC 0.5 ~ 15Å); (**B**) BG505 SOSIP.664 in complex with CH106 Fab (FSC 0.5 ~ 19Å); (**C**) BG505 SOSIP.664 in complex with 1NC9 Fab (FSC 0.5 ~ 18Å); (**D**) BG505 SOSIP.664 in complex with 3BNC117 Fab (FSC 0.5 ~ 20Å) and **(E)** BG505 SOSIP.664 in complex with VRC01 Fab (FSC 0.5 ~ 22Å).
(TIF)

S7 Fig Binding of 1NC9 and 8ANC195 to BG505 SOSIP.664 mutant trimers. (**A**) Representative binding curves for 2G12, VRC01, PGV04, CH103, 3BNC117, 1NC9 or 8ANC195 to the BG505 SOSIP.664 trimer and the D368R mutant. (**B**) Representative binding curves for 2G12, VRC01, PGV04, CH103, 3BNC117, 1NC9 or 8ANC195 to the BG505 SOSIP.664 trimer and N234S, N276S and N234S+N276S mutants.
(TIF)

## References

[1] Van Gils MJ, Sanders RW (2013) Broadly neutralizing antibodies against HIV-1: templates for a vaccine. Virology 435: 46–56. 10.1016/j.virol.2012.10.004 23217615

[2] Barouch DH, Whitney JB, Moldt B, Klein F, Oliveira TY, et al (2013) Therapeutic efficacy of potent neutralizing HIV-1-specific monoclonal antibodies in SHIV-infected rhesus monkeys. Nature 503: 224–228. 10.1038/nature12744 24172905 PMC4017780

[3] Van den Kerkhof TLGM, Euler Z, van Gils MJ, Boeser-Nunnink BD, Schuitemaker H, et al (2014) Early development of broadly reactive HIV-1 neutralizing activity in elite neutralizers. AIDS 28: 1237–1240. 10.1097/QAD.0000000000000228 24556870

[4] Doria-Rose N a, Schramm C a, Gorman J, Moore PL, Bhiman JN, et al (2014) Developmental pathway for potent V1V2-directed HIV-neutralizing antibodies. Nature 509: 55–62. 10.1038/nature13036 24590074 PMC4395007

[5] Blattner C, Lee JH, Sliepen K, Derking R, Falkowska E, et al (2014) Structural delineation of a quaternary, cleavage-dependent epitope at the gp41-gp120 interface on intact HIV-1 Env trimers. Immunity 40: 669–680. 10.1016/j.immuni.2014.04.008 24768348 PMC4057017

[6] Falkowska E, Le KM, Ramos A, Doores KJ, Lee JH, et al (2014) Broadly neutralizing HIV antibodies define a glycan-dependent epitope on the prefusion conformation of gp41 on cleaved envelope trimers. Immunity 40: 657–668. 10.1016/j.immuni.2014.04.009 24768347 PMC4070425

[7] West AP, Scharf L, Horwitz J, Klein F, Nussenzweig MC, et al (2013) Computational analysis of anti-HIV-1 antibody neutralization panel data to identify potential functional epitope residues. Proc Natl Acad Sci U S A 110: 10598–10603. 10.1073/pnas.1309215110 23754383 PMC3696754

[8] Huang J, Kang BH, Pancera M, Lee JH, Tong T, et al (2014) Broad and potent HIV-1 neutralization by a human antibody that binds the gp41-gp120 interface. Nature 515: 138–142. 10.1038/nature13601 25186731 PMC4224615

[9] Ringe RP, Sanders RW, Yasmeen A, Kim HJ, Lee JH, et al (2013) Cleavage strongly influences whether soluble HIV-1 envelope glycoprotein trimers adopt a native-like conformation. Proc Natl Acad Sci U S A 110: 18256–18261. 10.1073/pnas.1314351110 24145402 PMC3831437

[10] Klein F, Gaebler C, Mouquet H, Sather DN, Lehmann C, et al (2012) Broad neutralization by a combination of antibodies recognizing the CD4 binding site and a new conformational epitope on the HIV-1 envelope protein. J Exp Med 209: 1469–1479. 10.1084/jem.20120423 22826297 PMC3409500

[11] Lee JH et al. (2014) Pre-fusion gp41 conformational epitope antibodies neutralize HIV-1 by inducing viral spike decay. Submitted.

[12] Sanders RW, Derking R, Cupo A, Julien J-P, Yasmeen A, et al (2013) A next-generation cleaved, soluble HIV-1 Env Trimer, BG505 SOSIP.664 gp140, expresses multiple epitopes for broadly neutralizing but not non-neutralizing antibodies. PLoS Pathog 9: e1003618 10.1371/journal.ppat.1003618 24068931 PMC3777863

[13] Moore JP, Sodroski J (1996) Antibody cross-competition analysis of the human immunodeficiency virus type 1 gp120 exterior envelope glycoprotein. J Virol 70: 1863–1872. 8627711 10.1128/jvi.70.3.1863-1872.1996 PMC190014

[14] Julien J, Lee JH, Cupo A, Murin CD, Derking R, et al (2013) Asymmetric recognition of the HIV-1 trimer by broadly neutralizing antibody PG9. Proc Natl Acad Sci U S A 110: 4351–4356. 10.1073/pnas.1217537110 23426631 PMC3600498

[15] Binley JM, Sanders RW, Clas B, Schuelke N, Master a, et al (2000) A recombinant human immunodeficiency virus type 1 envelope glycoprotein complex stabilized by an intermolecular disulfide bond between the gp120 and gp41 subunits is an antigenic mimic of the trimeric virion-associated structure. J Virol 74: 627–643. 10623724 10.1128/jvi.74.2.627-643.2000 PMC111582

[16] Sanders RW, Vesanen M, Schuelke N, Master A, Schiffner L, et al (2002) Stabilization of the soluble, cleaved, trimeric form of the envelope glycoprotein complex of human immunodeficiency virus type 1. J Virol 76: 8875–8889. 12163607 10.1128/JVI.76.17.8875-8889.2002 PMC136973

[17] Julien J-P, Sok D, Khayat R, Lee JH, Doores KJ, et al (2013) Broadly neutralizing antibody PGT121 allosterically modulates CD4 binding via recognition of the HIV-1 gp120 V3 base and multiple surrounding glycans. PLoS Pathog 9: e1003342 10.1371/journal.ppat.1003342 23658524 PMC3642082

[18] Julien J-P, Cupo A, Sok D, Stanfield RL, Lyumkis D, et al (2013) Crystal structure of a soluble cleaved HIV-1 envelope trimer. Science 342: 1477–1483. 10.1126/science.1245625 24179159 PMC3886632

[19] Lyumkis D, Julien J-P, de Val N, Cupo A, Potter CS, et al (2013) Cryo-EM structure of a fully glycosylated soluble cleaved HIV-1 envelope trimer. Science 342: 1484–1490. 10.1126/science.1245627 24179160 PMC3954647

[20] Kong L, Lee JH, Doores KJ, Murin CD, Julien J-P, et al (2013) Supersite of immune vulnerability on the glycosylated face of HIV-1 envelope glycoprotein gp120. Nat Struct Mol Biol 20: 796–803. 10.1038/nsmb.2594 23708606 PMC3823233

[21] Pancera M, Zhou T, Druz A, Georgiev IS, Soto C, et al (2014) Structure and immune recognition of trimeric pre-fusion HIV-1 Env. Nature 514: 455–461. 10.1038/nature13808 25296255 PMC4348022

[22] Sanders RW, Moore JP (2014) HIV: A stamp on the envelope. Nature 514: 437–438. 10.1038/nature13926 25296251 PMC4836556

[23] Klasse PJ, Depetris RS, Pejchal R, Julien J-P, Khayat R, et al (2013) Influences on trimerization and aggregation of soluble, cleaved HIV-1 SOSIP envelope glycoprotein. J Virol 87: 9873–9885. 10.1128/JVI.01226-13 23824824 PMC3754145

[24] Khayat R, Lee JH, Julien J-P, Cupo A, Klasse PJ, et al (2013) Structural characterization of cleaved, soluble HIV-1 envelope glycoprotein trimers. J Virol 87: 9865–9872. 10.1128/JVI.01222-13 23824817 PMC3754114

[25] Pejchal R, Doores KJ, Walker LM, Khayat R, Huang P-S, et al (2011) A potent and broad neutralizing antibody recognizes and penetrates the HIV glycan shield. Science 334: 1097–1103. 10.1126/science.1213256 21998254 PMC3280215

[26] Scheid JF, Mouquet H, Ueberheide B, Diskin R, Klein F, et al (2011) Sequence and structural convergence of broad and potent HIV antibodies that mimic CD4 binding. Science 333: 1633–1637. 10.1126/science.1207227 21764753 PMC3351836

[27] Munro JB, Gorman J, Ma X, Zhou Z, Arthos J, et al (2014) Conformational dynamics of single HIV-1 envelope trimers on the surface of native virions. Science 346: 759–763. 10.1126/science.1254426 25298114 PMC4304640

[28] Guttman M, Cupo A, Julien J-P, Sanders RW, Wilson I a, et al (2015) Antibody potency relates to the ability to recognize the closed, pre-fusion form of HIV Env. Nat Commun 6: 6144 10.1038/ncomms7144 25652336 PMC4338595

[29] Hoffenberg S, Powell R, Carpov A, Wagner D, Wilson A, et al (2013) Identification of an HIV-1 clade A envelope that exhibits broad antigenicity and neutralization sensitivity and elicits antibodies targeting three distinct epitopes. J Virol 87: 5372–5383. 10.1128/JVI.02827-12 23468492 PMC3648150

[30] White T a, Bartesaghi A, Borgnia MJ, de la Cruz MJ V, Nandwani R, et al (2011) Three-dimensional structures of soluble CD4-bound states of trimeric simian immunodeficiency virus envelope glycoproteins determined by using cryo-electron tomography. J Virol 85: 12114–12123. 10.1128/JVI.05297-11 21937655 PMC3209358

[31] Walker LM, Huber M, Doores KJ, Falkowska E, Pejchal R, et al (2011) Broad neutralization coverage of HIV by multiple highly potent antibodies. Nature 477: 466–470. 10.1038/nature10373 21849977 PMC3393110

[32] Li Y, O’Dell S, Walker LM, Wu X, Guenaga J, et al (2011) Mechanism of neutralization by the broadly neutralizing HIV-1 monoclonal antibody VRC01. J Virol 85: 8954–8967. 10.1128/JVI.00754-11 21715490 PMC3165784

[33] Tran EEH, Borgnia MJ, Kuybeda O, Schauder DM, Bartesaghi A, et al (2012) Structural mechanism of trimeric HIV-1 envelope glycoprotein activation. PLoS Pathog 8: e1002797 10.1371/journal.ppat.1002797 22807678 PMC3395603

[34] Thali M, Moore JP, Furman C, Charles M, Ho DD, et al (1993) Characterization of conserved human immunodeficiency virus type 1 gp120 neutralization epitopes exposed upon gp120-CD4 binding. J Virol 67: 3978–3988. 7685405 10.1128/jvi.67.7.3978-3988.1993 PMC237765

[35] Yasmeen A, Ringe R, Derking R, Cupo A, Julien J-P, et al (2014) Differential binding of neutralizing and non-neutralizing antibodies to native-like soluble HIV-1 Env trimers, uncleaved Env proteins, and monomeric subunits. Retrovirology 11: 41 10.1186/1742-4690-11-41 24884783 PMC4067080

[36] Scharf L, Scheid JF, Lee JH, West AP, Chen C, et al (2014) Antibody 8ANC195 reveals a site of broad vulnerability on the HIV-1 envelope spike. Cell Rep 7: 785–795. 10.1016/j.celrep.2014.04.001 24767986 PMC4109818

[37] Zhou T, Georgiev I, Wu X, Yang Z-Y, Dai K, et al (2010) Structural basis for broad and potent neutralization of HIV-1 by antibody VRC01. Science 329: 811–817. 10.1126/science.1192819 20616231 PMC2981354

[38] Balla-Jhagjhoorsingh SS, Corti D, Heyndrickx L, Willems E, Vereecken K, et al (2013) The N276 glycosylation site is required for HIV-1 neutralization by the CD4 binding site specific HJ16 monoclonal antibody. PLoS One 8: e68863 10.1371/journal.pone.0068863 23874792 PMC3714269

[39] McGuire AT, Hoot S, Dreyer AM, Lippy A, Stuart A, et al (2013) Engineering HIV envelope protein to activate germline B cell receptors of broadly neutralizing anti-CD4 binding site antibodies. J Exp Med 210: 655–663. 10.1084/jem.20122824 23530120 PMC3620356

[40] Burton DR, Pyati J, Koduri R, Sharp SJ, Thornton GB, et al (1994) Efficient neutralization of primary isolates of HIV-1 by a recombinant human monoclonal antibody. Science 266: 1024–1027. 7973652 10.1126/science.7973652

[41] Trkola A, Pomales AB, Yuan H, Korber B, Maddon PJ, et al (1995) Cross-clade neutralization of primary isolates of human immunodeficiency virus type 1 by human monoclonal antibodies and tetrameric CD4-IgG. J Virol 69: 6609–6617. 7474069 10.1128/jvi.69.11.6609-6617.1995 PMC189569

[42] Muster T, Steindl F, Purtscher M, Trkola a, Klima a, et al (1993) A conserved neutralizing epitope on gp41 of human immunodeficiency virus type 1. J Virol 67: 6642–6647. 7692082 10.1128/jvi.67.11.6642-6647.1993 PMC238102

[43] Zwick MB, Labrijn AF, Wang M, Spenlehauer C, Saphire EO, et al (2001) Broadly neutralizing antibodies targeted to the membrane-proximal external region of human immunodeficiency virus type 1 glycoprotein gp41. J Virol 75: 10892–10905. 11602729 10.1128/JVI.75.22.10892-10905.2001 PMC114669

[44] Wibmer CK, Bhiman JN, Gray ES, Tumba N, Abdool Karim SS, et al (2013) Viral escape from HIV-1 neutralizing antibodies drives increased plasma neutralization breadth through sequential recognition of multiple epitopes and immunotypes. PLoS Pathog 9: e1003738 10.1371/journal.ppat.1003738 24204277 PMC3814426

[45] Gao F, Bonsignori M, Liao H-X, Kumar A, Xia S-M, et al (2014) Cooperation of B cell lineages in induction of HIV-1-broadly neutralizing antibodies. Cell 158: 481–491. 10.1016/j.cell.2014.06.022 25065977 PMC4150607

[46] Carrillo J. et al (n.d.) Gp120/CD4 Blocking Antibodies are Frequently Elicited in ART-naïve Chronically HIV-1 Infected Individuals. Submitted. 10.1371/journal.pone.0120648 PMC4372395 25803681

[47] Voss NR, Yoshioka CK, Radermacher M, Potter CS, Carragher B (2009) DoG Picker and TiltPicker: software tools to facilitate particle selection in single particle electron microscopy. J Struct Biol 166: 205–213. 19374019 10.1016/j.jsb.2009.01.004 PMC2768396

[48] Ogura T, Iwasaki K, Sato C (2003) Topology representing network enables highly accurate classification of protein images taken by cryo electron-microscope without masking. J Struct Biol 143: 185–200. 14572474 10.1016/j.jsb.2003.08.005

[49] Lander GC, Stagg SM, Voss NR, Cheng A, Fellmann D, et al (2009) Appion: an integrated, database-driven pipeline to facilitate EM image processing. J Struct Biol 166: 95–102. 19263523 10.1016/j.jsb.2009.01.002 PMC2775544

[50] Tang G, Peng L, Baldwin PR, Mann DS, Jiang W, et al (2007) EMAN2: an extensible image processing suite for electron microscopy. J Struct Biol 157: 38–46. 16859925 10.1016/j.jsb.2006.05.009

[51] Ludtke SJ, Baldwin PR, Chiu W (1999) EMAN: semiautomated software for high-resolution single-particle reconstructions. J Struct Biol 128: 82–97. 10600563 10.1006/jsbi.1999.4174

[52] Pettersen EF, Goddard TD, Huang CC, Couch GS, Greenblatt DM, et al (2004) UCSF Chimera—a visualization system for exploratory research and analysis. J Comput Chem 25: 1605–1612. 15264254 10.1002/jcc.20084

