# Supplementary figures and images for: Comprehensive Antigenic Map of a Cleaved Soluble HIV-1 Envelope Trimer
Source: PLoS Pathog. 2015 Mar 25;11(3):e1004767. doi: 10.1371/journal.ppat.1004767 (PMC4373910; doi:10.1371/journal.ppat.1004767)

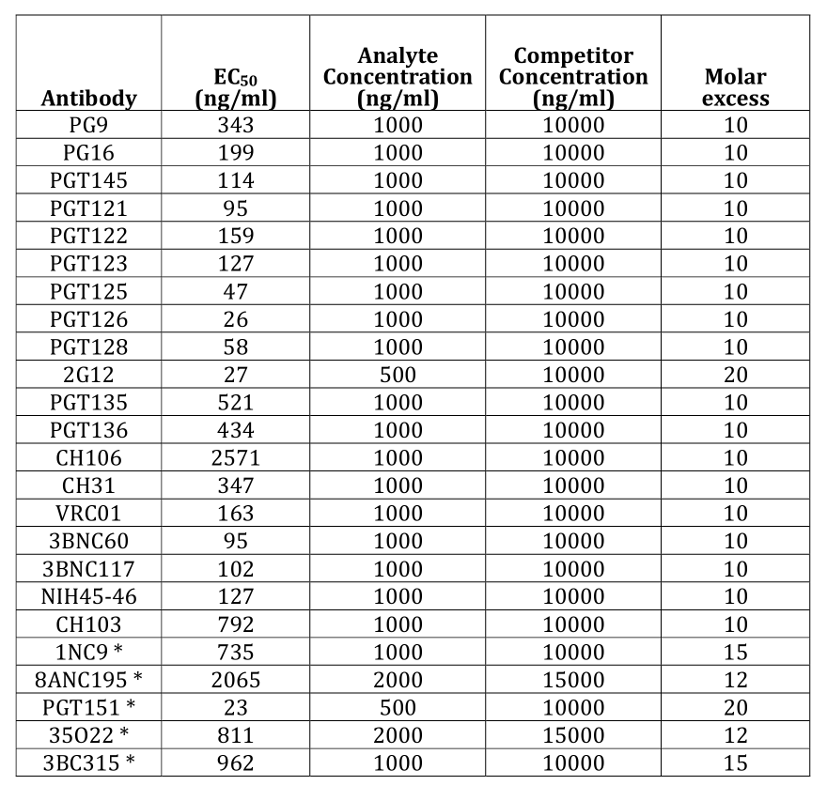

Supplement: S1 Table — Midpoint binding titers (EC50 values; second column) are taken from reference [12]. The analyte and competitor concentrations used here are given in the third and fourth column, and the molar ratio between the analyte and competitor antibodies are listed in the last column. Note that the molar ratios for Fab competitors (see asterisks) were halved to reflect the monovalency of the combining site compared to IgG. (TIF) [file ppat.1004767.s001.tif]

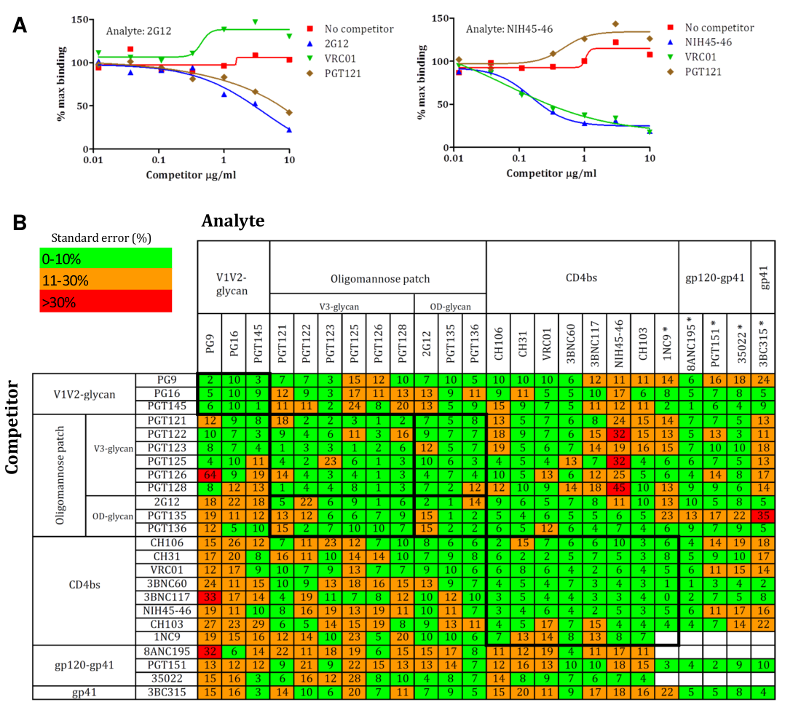

Supplement: S1 Fig — (A) Detection of biotinylated versions of 2G12 or NIH45-46 in the presence of escalating amounts of IgG competitors. (B) The extent of the error between individual data points is depicted by color-coding: Green, orange and red indicate small, intermediate and large standard errors between the individual data points, respectively. (TIF) [file ppat.1004767.s002.tif]

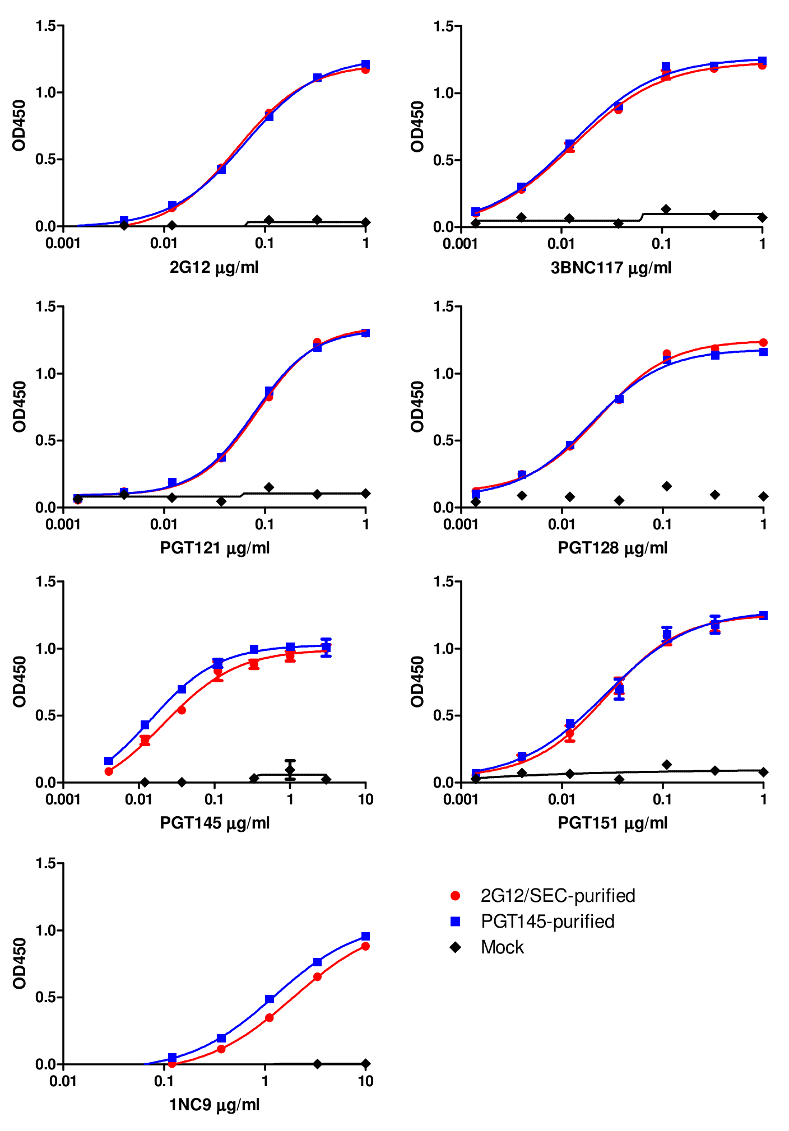

Supplement: S2 Fig — The 2G12 bNAb was used as a loading control and 1NC9 and PGT151 were used to probe the allosteric changes are still present in the PGT145 purified trimers. Because the binding of 1NC9 and PGT151 to 1G12/SEC- and PGT145-purified trimer is similar, we infer that the PGT145-induced allosteric changes that inhibit 1NC9 and PGT151 binding (Fig. 1) are reversible. (TIF) [file ppat.1004767.s003.tif]

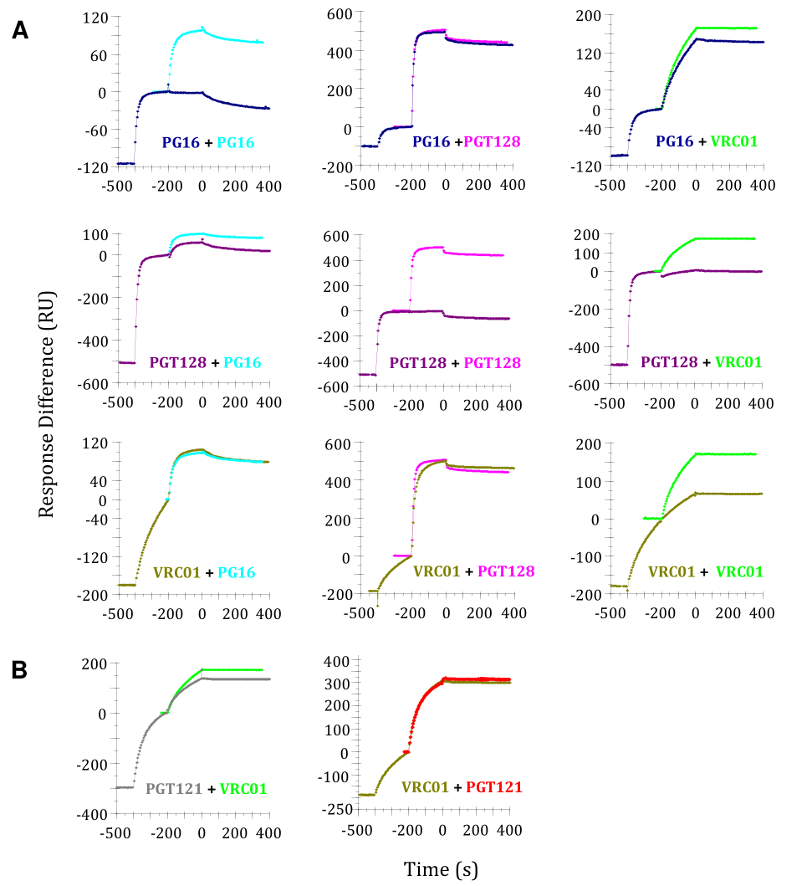

Supplement: S3 Fig — (A) Competitions between PG16, PGT128 and VRC01. Association-dissociation curves of the individual binding experiments were overlaid with the second association phase to detect competition. 0 on the y axis is the baseline for the single comparator injection and for the same analyte as the second in the double injection. Thus, all three responses can be read on the same scale, although the value for the first analyte in the double injection will be negative. (B) Competition between PGT121 and VRC01. (TIF) [file ppat.1004767.s004.tif]

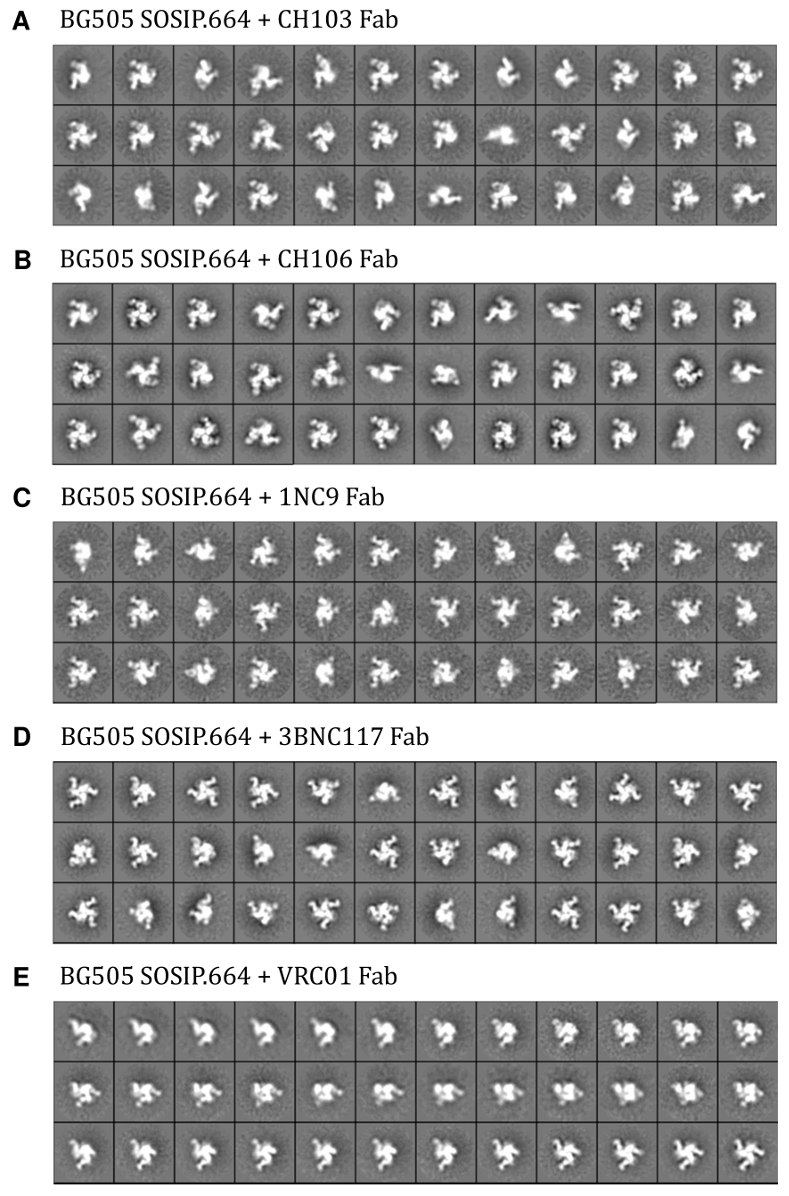

Supplement: S4 Fig — Shown are the 2D class-averages of complexes of the trimers with (A) CH103 Fabs; (B) CH106 Fabs; (C) 1NC9 Fabs; (D) 3BNC117 Fabs; and (E) VRC01 Fabs. For reconstructions of the unliganded BG505 SOSIP.664 trimer, see references [12,14]. (TIF) [file ppat.1004767.s005.tif]

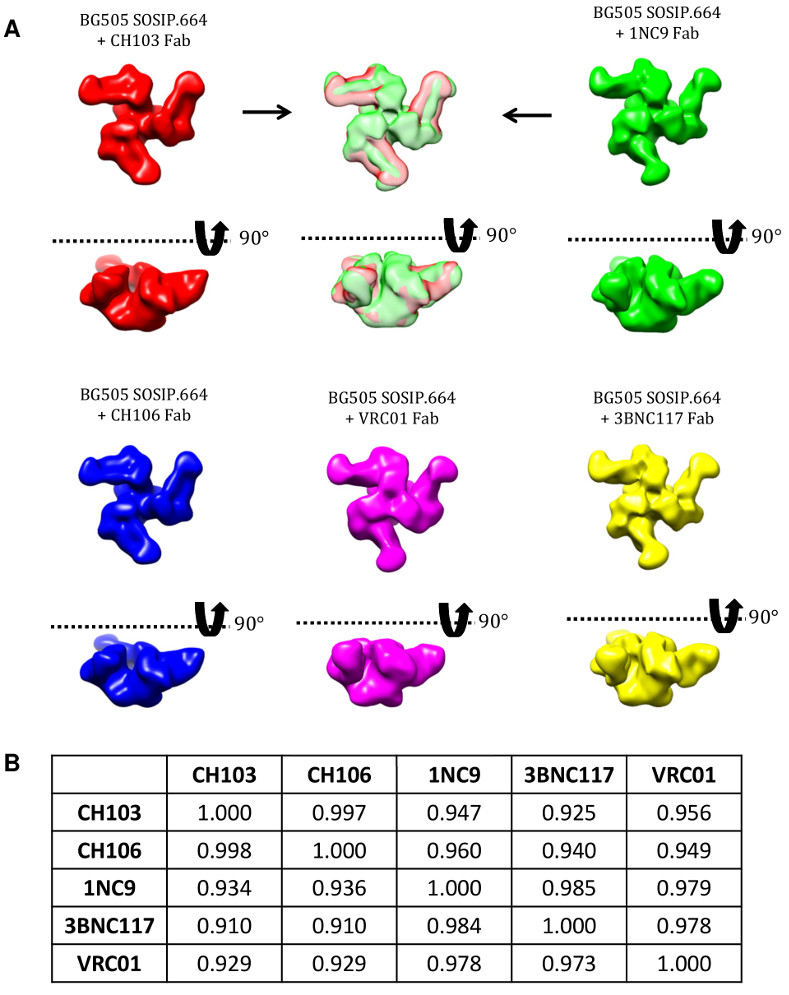

Supplement: S5 Fig — (A) EM reconstruction of trimers in complex with CH103, CH106, 1NC9 3BNC117 and VRC01 Fabs, with a fit of the CH103 complex map into the 1NC9 complex map shown at center. (B) Cross-correlation coefficients from map fitting of the four BG505 SOSIP.664-Fab complexes. (TIF) [file ppat.1004767.s006.tif]

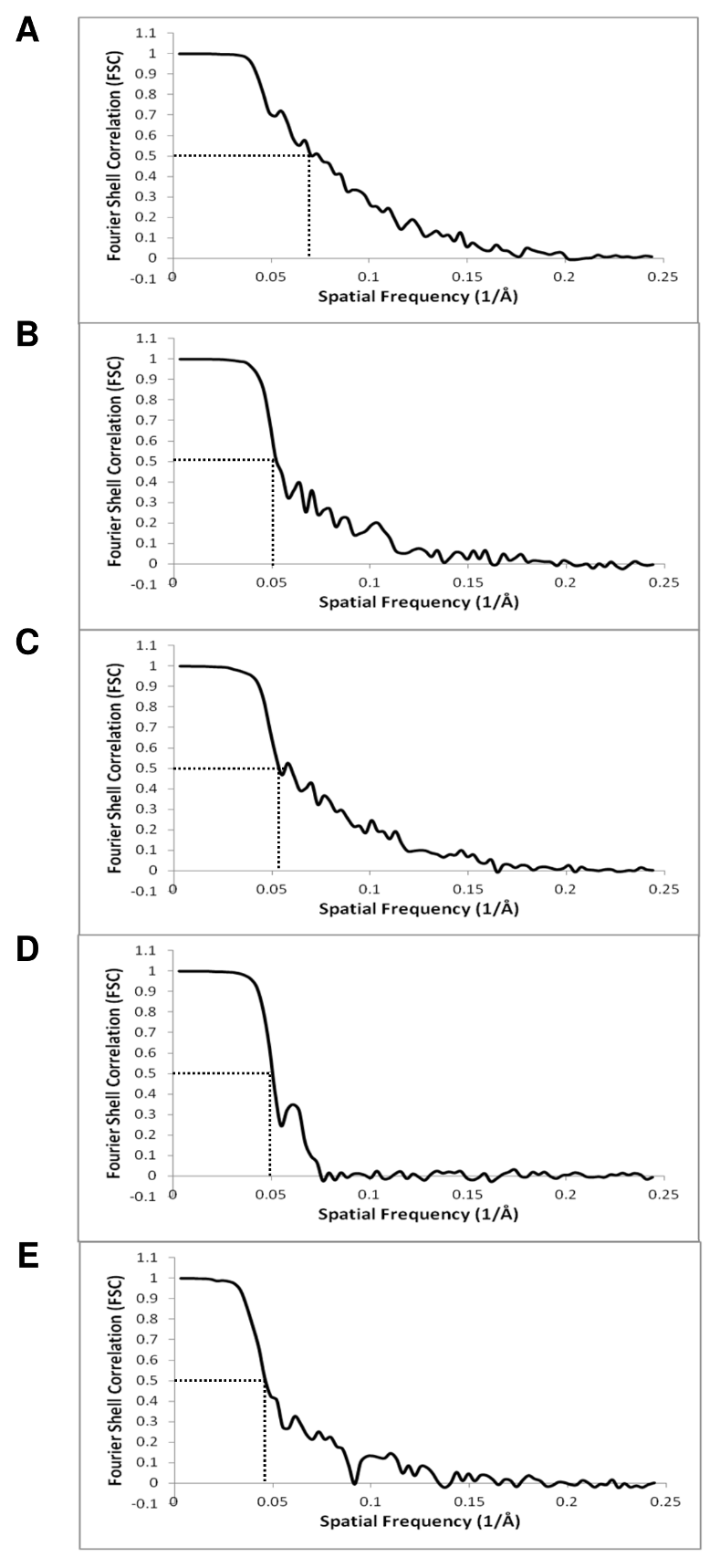

Supplement: S6 Fig — (A) BG505 SOSIP.664 in complex with CH103 Fab (FSC 0.5 ~ 15Å); (B) BG505 SOSIP.664 in complex with CH106 Fab (FSC 0.5 ~ 19Å); (C) BG505 SOSIP.664 in complex with 1NC9 Fab (FSC 0.5 ~ 18Å); (D) BG505 SOSIP.664 in complex with 3BNC117 Fab (FSC 0.5 ~ 20Å) and (E) BG505 SOSIP.664 in complex with VRC01 Fab (FSC 0.5 ~ 22Å). (TIF) [file ppat.1004767.s007.tif]

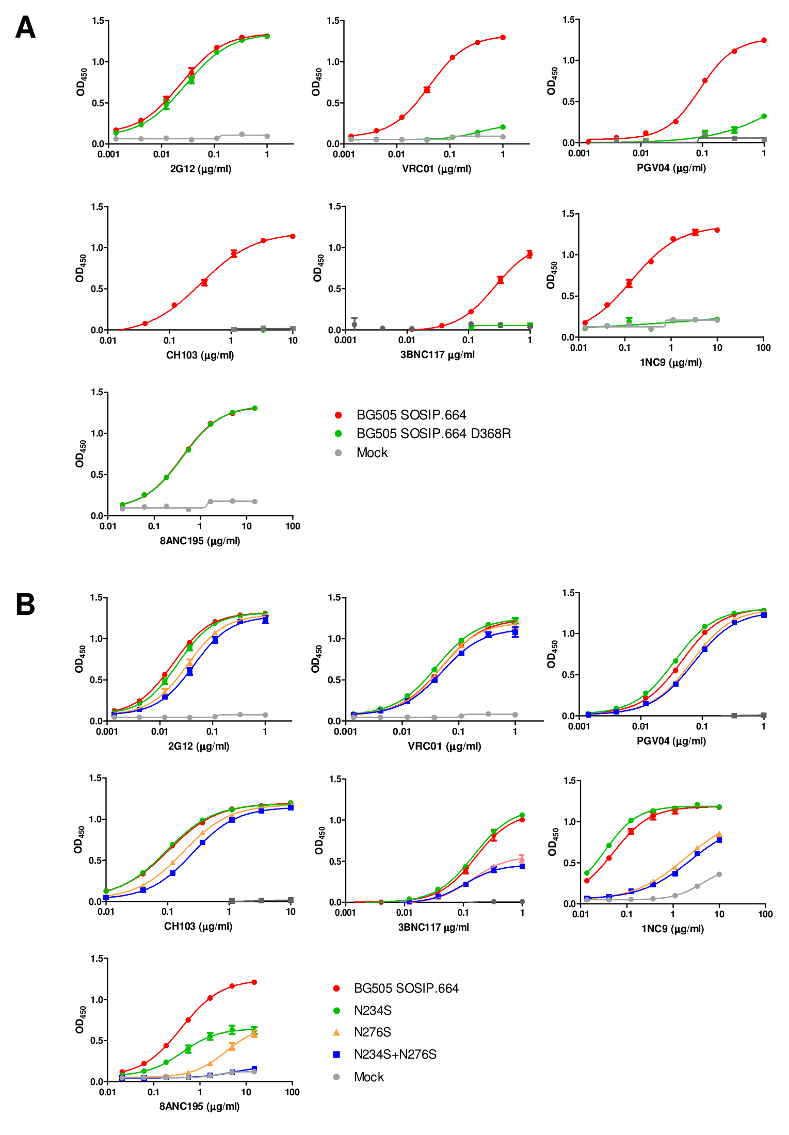

Supplement: S7 Fig — (A) Representative binding curves for 2G12, VRC01, PGV04, CH103, 3BNC117, 1NC9 or 8ANC195 to the BG505 SOSIP.664 trimer and the D368R mutant. (B) Representative binding curves for 2G12, VRC01, PGV04, CH103, 3BNC117, 1NC9 or 8ANC195 to the BG505 SOSIP.664 trimer and N234S, N276S and N234S+N276S mutants. (TIF) [file ppat.1004767.s008.tif]
